# A Three Amino Acid Sequence Gates Protein Stability Control of bHLH104 and Iron Uptake in *Arabidopsis thaliana*

**DOI:** 10.1111/pce.70879

**Published:** 2026-09-22

**Authors:** Dibin Baby, Sarah Weldi, Marie Knopf, Vinitha Venkadasamy, Jessica Lee Erickson, Petra Bauer

**Affiliations:** ^1^ Institute of Botany, Heinrich Heine University Düsseldorf Germany; ^2^ Cluster of Excellence on Plant Science (CEPLAS) Heinrich Heine University Düsseldorf Germany; ^3^ Zentrum für Molekularbiologie der Pflanzen (ZMBP), Allgemeine Genetik University of Tübingen Tübingen Germany

**Keywords:** *Arabidopsis thaliana*, bHLH IVc, BTS, iron, transcription factor, ubiquitination

## Abstract

Iron (Fe) homeostasis is regulated to prevent iron imbalance, with the help of redundant basic helix‐loop‐helix (bHLH) IVc transcription factors that can be controlled by Fe‐binding E3 ligases such as BRUTUS (BTS) in *Arabidopsis thaliana*. However, knowledge gaps remain to fully explain the mechanistic basis of this redundant protein interaction module. The C‐terminus of bHLH104 is known to interact with BTS, and structural predictions implicate the three terminal amino acids, proline‐alanine‐alanine (PAA). However, the importance of the PAA sequence for post‐translational regulation of bHLH104 and for the resulting plant phenotypes has not been tested experimentally. Here we show that transgenic plants expressing a bHLH104 variant lacking PAA (b104^dPAA) constitutively upregulate Fe acquisition and Fe transport genes and accumulate Fe, whereas plants expressing wild‐type bHLH104 do not. The extent of Fe accumulation in independent b104^dPAA lines correlated positively with b104^dPAA protein abundance. Unlike the wild‐type form, b104^dPAA showed no indication of interaction with BTS or of ubiquitination by it. Together, these findings support a model in which the PAA sequence serves as a recognition motif for binding to BTS and for subsequent post‐translational regulation of bHLH104. Hence, targeted manipulation of the bHLH IVc protein PAA sequence may represent a strategy for crop biofortification.

## Summary statement

Three C‐terminal amino acids gate the stability of the transcription factor bHLH104 in *Arabidopsis thaliana*. Their removal stabilises the protein and drives constitutive iron uptake, increasing iron content at both the seedling and the reproductive stage.

## 1 Introduction

Iron (Fe) is an essential micronutrient required for plant growth and development. It plays critical roles in photosynthesis, respiration, chlorophyll biosynthesis, and redox reactions (Briat et al. 2007; Jeong and Guerinot 2009). Although Fe is abundant in the Earth's crust, it is mostly present in the ferric (Fe^3+^) form, which is poorly soluble and difficult for plant roots to absorb, especially in alkaline or calcareous soils (Marschner 2012). Excess Fe is toxic since it generates reactive oxygen species (Panda and Yamamoto 2019). Therefore, plants must tightly regulate Fe uptake, distribution, and storage to maintain internal balance.

In *Arabidopsis thaliana*, a group of basic helix–loop–helix (bHLH) IVc transcription factors, including bHLH34, bHLH104, ILR3 (bHLH105), and bHLH115, acts as an early regulatory module in the Fe deficiency (−Fe) response. These proteins activate the expression of internal Fe allocation and Fe deficiency response regulatory genes in roots and shoots (Schwarz and Bauer 2020). Among their targets are also genes coding for bHLH Ib transcription factors that, together with the bHLH transcription factor FIT (FER‐LIKE IRON DEFICIENCY‐INDUCED TRANSCRIPTION FACTOR), induce root Fe uptake genes such as *IRT1* and *FRO2* (Long et al. 2010; Selote et al. 2015; Zhang et al. 2015; Li et al. 2016; Liang et al. 2017).

The bHLH IVc transcription factors are regulated predominantly at the level of protein abundance. Transcript levels of *BHLH IVc* genes do not change substantially under −Fe conditions (Zhang et al. 2015; Li et al. 2016; Liang et al. 2017), indicating that they are activated during the −Fe response primarily at the protein level. It is proposed that bHLH IVc proteins are subject to post‐translational regulation via the 26S proteasome, mediated by the E3 ubiquitin ligases BRUTUS (BTS) and BTS‐LIKE (BTSL) proteins (Zhao et al. 2026). bHLH105/ILR3 and bHLH115 have been shown to be recognised, ubiquitinated and degraded by BTS and BTSL proteins (Selote et al. 2015; Hindt et al. 2017; Zhao et al. 2026), but this has not yet been demonstrated experimentally for all bHLH IVc proteins. Control by E3 ligases of the BTS/BTSL‐type is particularly intriguing since stability of the BTS/BTSL E3 ligases is controlled by their N‐terminus, which contains hemerythrin domains for Fe and oxygen sensing (Selote et al. 2015; Pullin et al. 2025). Additionally, Fe and Zn binding to C‐terminal zinc finger domains (CHY, CTCHY, RING) and rubredoxin‐type folds of the BTS rice ortholog HEMERYTHRIN MOTIF‐CONTAINING REALLY INTERESTING NEW GENE AND ZINC‐FINGER PROTEIN (HRZ) may regulate its protein stability and activity, suggesting an additional mechanism for metal ion sensing (Shinkawa et al. 2025). Together, these findings support a model in which, under Fe deficiency, altered metal occupancy and redox state of BTS/BTSL domains attenuate E3 ligase activity toward bHLH IVc transcription factors, allowing accumulation of the transcription factors and subsequent activation of Fe uptake genes. This model is further supported by genetic studies demonstrating that loss of *BTS* (Selote et al. 2015; Long et al. 2010) or *BTSL1/BTSL2* (Hindt et al. 2017; Rodríguez‐Celma et al. 2019) results in activation of Fe deficiency responses.

One bHLH IVc group protein is bHLH104 (Zhang et al. 2015; Li et al. 2016). Phylogenetic analysis places bHLH104 together with bHLH34 in a distinct clade from bHLH105 and bHLH115, and individual bHLH IVc members show distinct expression patterns (Gao et al. 2019, 2024; Jiang et al. 2024), indicating that regulatory mechanisms cannot be directly extrapolated between members. T‐DNA loss‐of‐function lines of *bHLH104* exhibit an Fe deficiency‐sensitive phenotype (Li et al. 2016), which is expected for an upstream regulator of the Fe deficiency response. Genetic evidence places bHLH104 downstream of BTS, as loss of bHLH104 partially suppresses the Fe deficiency tolerance of the *bts‐2* mutant (Zhang et al. 2015). The C‐terminus of bHLH104 can interact with BTS/L proteins (Lichtblau et al. 2022). It possesses the sequence PAA at the very C‐terminal end. In our previous study, we showed that deleting a 25‐amino‐acid‐long stretch at the C terminus abolished bHLH104 interaction with BTS (Lichtblau et al. 2022). Other studies implicated the terminal residue of this C‐terminal region in the regulation of bHLH IVc proteins (Sharma and Yeh 2020; Li et al. 2021; Zhao et al. 2026). Li et al. (2021) demonstrated that substitution of the terminal alanine with valine in bHLH105 and bHLH115 abolished interaction with BTS in yeast two‐hybrid (Y2H) assays and increased protein stability in transient expression assays. Sharma and Yeh (2020) identified an ethyl methanesulfonate‐induced C‐terminal alanine‐to‐valine substitution in bHLH34 that conferred increased protein stability and metal accumulation in plants. Zhao et al. (2026) subsequently showed by Y2H assay that substitution of the terminal alanine by valine abolishes the interaction with BTS for all four *Arabidopsis* bHLH IVc proteins, establishing the terminal residue as a determinant of BTS recognition.

A C‐terminal sequence with terminal PAA is conserved in IRON MAN (IMA)/FE‐UPTAKE‐INDUCING PEPTIDE (FEP) small proteins. Lichtblau et al. (2022) found that the C‐terminal portion of IMA1 small proteins is needed for IMA1 to interact with the BTSL1 C‐terminus. Moreover, they described that intact IMA1, but not IMA1 with a C‐terminal deletion, disturbed We like to exchange the Figure

The role of the bHLH104 PAA sequence for protein stability and function in planta has not been examined. It is also not known whether bHLH104 is a substrate of BTS‐mediated ubiquitination, as has been shown for bHLH105 and bHLH115 (Li et al. 2021; Zhao et al. 2026). In this study, we deleted the three C‐terminal residues of bHLH104 and expressed the variant under the native *BHLH104* promoter in *A. thaliana*. We examined whether the deletion causes Fe‐related growth phenotypes, whether these phenotypes can be attributed to altered protein abundance, and whether the PAA sequence is required for interaction with BTS and for BTS‐mediated ubiquitination. Transgenic lines were analysed at the seedling and reproductive stages.

## 2 Results

### 2.1 Plants With bHLH104 Lacking PAA (*b104^dPAA*) Have Distinct Growth Phenotypes

We hypothesised that the PAA sequence plays a role in the functionality of bHLH104 in plants. To test this, we studied transgenic *A. thaliana* lines in the WT Col‐0 background expressing either regular full‐length mTurquoise2 fluorophore‐tagged bHLH104 (hereafter *pb104::b104*) or a mutant deletion variant lacking the conserved PAA sequence (hereafter *pb104::b104^dPAA*), both driven by the native *BHLH104* promoter (Figure 1A). The N‐terminal mTurquoise2‐tagged b104^dPAA complemented the *bhlh104‐1* seedling −Fe sensitivity phenotype, indicating functionality of the fusion protein (Supporting Information S1: Figure S1).

**Figure 1:**
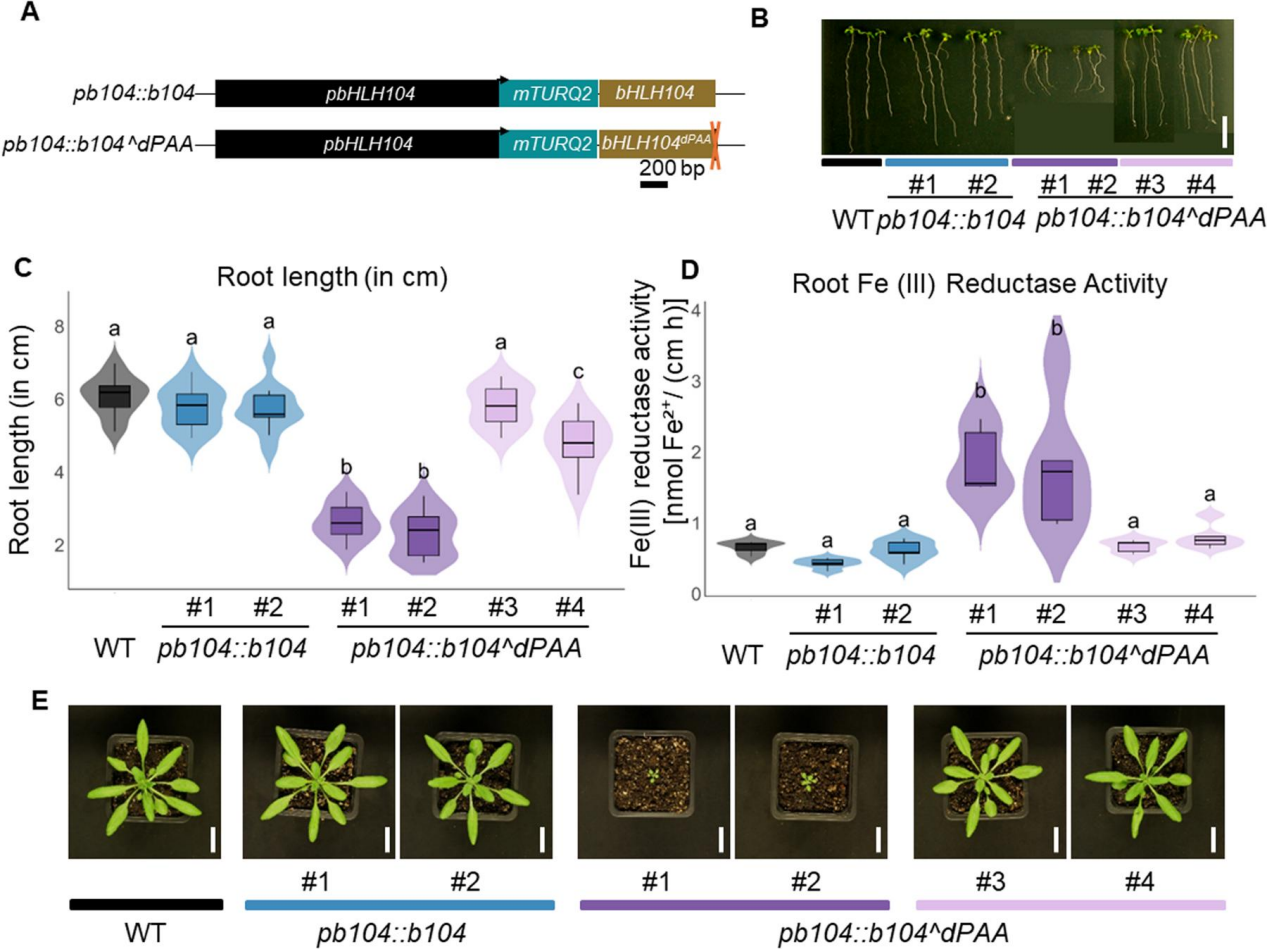
Transgenic expression of bHLH104 lacking the PAA sequence leads to growth alterations and physiological changes in *Arabidopsis*. (A) Schematic representation of constructs used to generate transgenic lines expressing either full‐length bHLH104 (*pb104::b104*) or a C‐terminal deletion variant lacking the conserved three–amino acid PAA sequence (*pb104::b104^dPAA*). Both constructs are driven by the native *BHLH104* promoter (1636 bp) and fused at the N‐terminus to an mTurquoise2 fluorescent tag. The deleted PAA sequence in b104^dPAA is indicated by a red cross at the C‐terminus. Scale bar = 200 bp. (B) Representative images of 10‐day‐old seedlings grown on +Fe medium (scale bar = 2 cm). (C) Primary root length of 12‐day‐old seedlings (*n* = 12) grown vertically on Hoagland medium supplemented with Fe. (D) Fe reductase activity in roots of 10‐day‐old seedlings grown under +Fe. Activity was quantified spectrophotometrically as Fe(II)–ferrozine complex formation and normalised to total root length. Data represent three biological replicates (*n* = 3). (C, D) Different letters indicate significant differences between genotypes (two‐way ANOVA followed by Tukey's HSD test, *p* < 0.05). (E) Representative images of 4‐week‐old soil‐grown plants (scale bar = 2 cm). Shown are Col‐0 (WT), two independent *pb104::b104* lines (#1, #2), and four independent *pb104::b104^dPAA* lines (#1–#4).

Phenotypic variation was visible in the T1 population of the *pb104::b104^dPAA* transgenics. While the majority of T1 plants displayed severely reduced growth with brown necrotic spots on leaves, some plants appeared indistinguishable from wild type (WT) (Supporting Information S2: Figure S2).

To examine the basis of this variation, we quantified relative transgene copy number, transgene transcript level, b104^dPAA protein abundance and iron content across the eight independent *pb104::b104^dPAA* lines and related them to primary root length as a measure of phenotype severity (Supporting Information S3: Figure S3). Phenotype severity correlated with transgene copy number, b104^dPAA protein and iron content, but not with transgene transcript level, indicating that the variation between lines reflects b104^dPAA protein abundance rather than transgene silencing or the site of insertion.

Four lines were selected for detailed morphological and physiological characterisation across the life cycle, two with severe phenotypes (#1, #2) and two with no obvious macroscopic phenotype *(#3, #4*), in addition to *pb104::b104 #1* and *#2* controls. The assignment of lines to each analysis is summarised in Supporting Information S2: Figure S2B.

To quantify phenotypic variation across the life cycle, detailed morphological and physiological assessments were made with T3 plants selected for transgene presence by genotyping PCR and grown on agar plates or soil. In 12‐day‐old seedlings of *pb104::b104^dPAA* lines *#1* and *#2*, we found significantly shorter primary roots than in seedlings of lines #3 and #4 or in pb104::b104 control lines (Figure 1B,C). Root Fe(III) reductase activity is necessary for reduction‐based Strategy I Fe uptake (Yi and Guerinot 1996). Ferric reductase activity measurements revealed that *pb104::b104^dPAA* lines *#1* and *#2* had higher Fe reductase activity compared to lines #3 and #4 and the controls (Figure 1D). The Phenospex PlantEye phenotyping platform detects small differences in leaf colour and in 3D leaf area, defined as the leaf surface area derived from the three‐dimensional reconstruction of the plant (see Section 4), in *A. thaliana* (Knopf and Bauer 2025). By 4 weeks, *pb104::b104^dPAA* lines *#1* and *#2* displayed smaller rosette area (Figure 1E; Supporting Information S4: Figure S4A) and lower Normalised Difference Vegetation Index (NDVI) values (Supporting Information S4: Figure S4B), which indicate lower chlorophyll content, lower photosynthetic efficiency, and reduced growth performance compared with other lines. Flowering was delayed compared to the other lines (Supporting Information S4: Figure S4C), and seed yield was significantly lower for *pb104::b104^dPAA* lines *#1* and *#2* compared to the other lines (Supporting Information S4: Figure S4D).

Reduced root and shoot growth, as observed in plants of *pb104::b104^dPAA* line #2, can be a consequence of Fe toxicity (Naranjo‐Arcos et al. 2017). Thus, we tested whether the growth defects observed in the *pb104::b104^dPAA line #*2 plants were alleviated in conditions of Fe deficiency. We grew the representative lines on alkaline calcareous soil (ACS; pH 8.1), which provides Fe‐limiting conditions (Supporting Information S5: Figure S5; Knopf and Bauer 2025). Notably, the *pb104::b104^dPAA line #2*, which exhibited pronounced growth defects in control soil (pH 5.9), showed increased 3D leaf area in ACS, indicating partial recovery under Fe‐limited ACS conditions (Supporting Information S5: Figure S5C). NDVI values, which were low in control soil, were restored to near‐WT levels in ACS, suggesting improved leaf health (Supporting Information S5: Figure S5D). While flowering remained delayed relative to WT, it occurred earlier in ACS than in control soil (Supporting Information S5: Figure S5E). These findings show that the growth defects observed in control soil are largely alleviated when Fe availability is limited, linking the b104^dPAA‐associated phenotypes to Fe status.

Together, these results show that removal of the PAA sequence in bHLH104 can lead to dominant growth phenotypes across the life cycle. To explore whether these phenotypes reflect altered Fe homeostasis, we next examined tissue Fe content and expression of Fe‐responsive genes in both seedlings and plants at the reproductive stage. For further detailed experiments, we focused on two representative *pb104::b104^dPAA* lines, namely a strong line, #2, and a weak phenotype line, #3.

### 2.2 b104^dPAA Can Cause Constitutive Fe Uptake and Transcriptional Misregulation of Fe Deficiency Response Genes in Seedlings

After a few days of germination, *A. thaliana* seedlings rely on external Fe supply. To assess whether b104^dPAA affects Fe homeostasis during early development, we measured total Fe content via inductively coupled plasma‐mass spectroscopy (ICP‐MS) and analysed gene expression profiles in 10‐day‐old seedlings of WT, control and contrasting *pb104::b104^dPAA* lines #2 and #3 in both +Fe and −Fe conditions (Figure 2A,B). As an additional control, we used *pUBQ10::b104* lines expressing full‐length bHLH104 constitutively from the strong *UBIQUITIN10* promoter, allowing us to assess whether transcript levels alone affect Fe homeostasis. Morphologically, *pUBQ10::b104* lines #1 and #2 are indistinguishable from WT, except for slightly shorter roots (Supporting Information S6: Figure S6).

**Figure 2:**
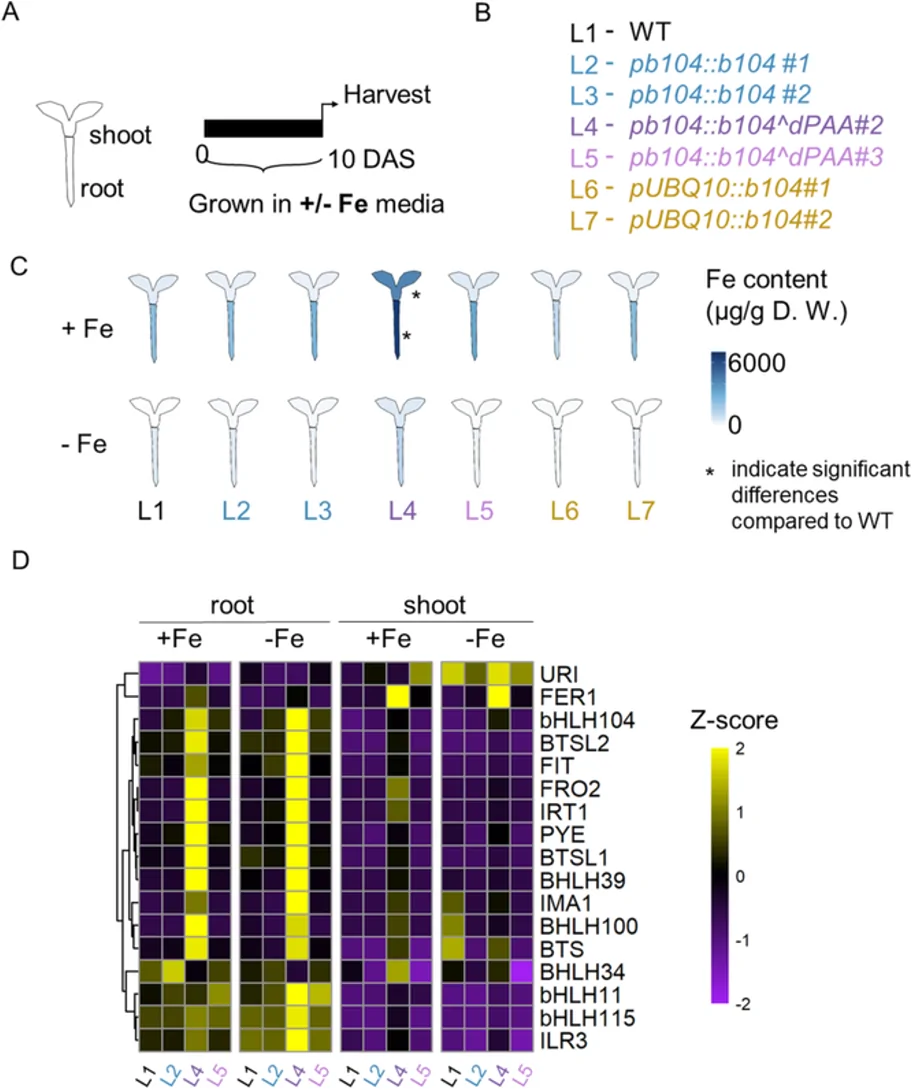
Transgenic lines expressing b104^dPAA exhibit increased iron accumulation and altered iron homeostasis gene expression in the seedling stage. (A) Schematic representation of the experimental design. *Arabidopsis* seedlings were grown for 10 days after sowing (DAS) under either iron‐sufficient (+Fe) or iron‐deficient (−Fe) conditions. Shoots and root tissues were collected separately for ICP‐MS and gene expression analyses. (B) Genotypes used in this study. (C) Iron content in shoot and root tissues of seedlings grown under +Fe and −Fe conditions, measured by ICP‐MS. Data represent the mean of three biological replicates (*n* = 3). Heatmap intensity reflects iron concentration (µg/g dry weight). Colour shading was rendered using the R package ggplantmap, with darker blue indicating higher Fe accumulation. Notably, *pb104::b104^dPAA* lines show increased Fe accumulation in roots and shoots under +Fe conditions. Asterisks indicate genotypes that differ significantly from WT within the same tissue and treatment condition (*p* < 0.05), as determined by two‐way ANOVA and Tukey's post hoc test (*n* = 3 for each group). (D) Clustered heatmaps showing the expression profiles (RNA sequencing) of selected Fe homeostasis genes across genotypes, tissues (root and shoot) and treatments (+Fe and −Fe). Heatmaps were generated in RStudio version 4.4.1 using *Z*‐scores of normalised gene expression values calculated across all samples (*n* = 3 for each group). Purple indicates low expression, and yellow indicates high expression. Because *Z*‐scores are calculated across all samples to convey relative expression patterns, absolute expression magnitudes, including differences between root and shoot and between treatments, are not directly comparable within this representation. Expression data of canonical Fe‐deficiency marker genes in this dataset is shown in Supporting Information S22: Figure S22. [Color figure can be viewed at wileyonlinelibrary.com]

Elemental profiling revealed that the *pb104::b104^dPAA* line #2 accumulated substantially more Fe than all other genotypes under both +Fe and −Fe conditions (Figure 2C). This increase was observed in both roots and shoots, with roots showing higher Fe levels than shoots. In contrast, the *pb104::b104^dPAA* line #3 and the *pb104::b104* and *pUBQ10::b104* lines had Fe contents similar to WT. All lines exhibited reduced Fe contents under −Fe, confirming the effectiveness of the −Fe treatment (Figure 2C).

Differences were also detected for manganese (Mn), zinc (Zn), and copper (Cu) levels (Supporting Information S7: Figure S7). Uptake of these metal ions is closely connected with Fe homeostasis (Connolly et al. 2002). In *pb104::b104^dPAA* line #2, Zn and Cu were elevated in roots under −Fe, and Cu was increased in shoots. Notably, Zn levels were elevated even under +Fe in *pb104::b104^dPAA* roots compared to WT. Roots of *pUBQ10::b104* lines also showed increased Mn under −Fe compared to WT. Metal concentrations in the other lines were similar to those of WT. Hence, these findings suggest that the *pb104::b104^dPAA* line #2 has increased Fe accumulation and affects the uptake of other micronutrients such as Zn and Cu. This highlights the importance of proper regulation of bHLH104 function in maintaining metal homeostasis under varying Fe conditions.

To elucidate the transcriptional response, we analysed gene expression patterns via RNA sequencing (RNA‐seq) following −Fe treatment at the seedling stage in both root and shoot tissues. The highest number of differentially expressed genes (DEGs) was observed in the shoot tissue of *pb104::b104^dPAA line #2*, with 4645 DEGs under +Fe conditions and 2857 DEGs under −Fe conditions after 10 days of treatment, compared to WT. There was also substantial differential expression in roots, with 1210 DEGs under +Fe conditions and 111 DEGs under −Fe conditions (Supporting Information S8: Figure S8A).

Gene Ontology (GO) analysis revealed that the upregulated genes in the shoot tissue of the *pb104::b104^dPAA line #*2 plants were enriched in terms related to hypoxia and oxidative stress, while genes associated with photosynthesis were downregulated. In *pb104::b104^dPAA* root tissue, upregulated gene categories were additionally enriched for terms related to metal ion transport, while downregulated categories showed enrichment for cell wall modification‐related GO terms (Supporting Information S8: Figure S8B). Hence, the *pb104::b104^dPAA line #*2 plants indeed show symptoms of Fe overload with associated oxidative stress. These enriched GO terms most likely reflect secondary responses to Fe and metal overload.

Gene expression profiles can inform on misregulation in mutants and transgenic lines (Schwarz and Bauer 2020). We examined the expression patterns of selected genes involved in Fe uptake and homeostasis regulation to characterise their specific regulatory expression profiles. Fe acquisition and mobilisation genes were constitutively upregulated in *pb104::b104^dPAA* line #2 regardless of Fe availability at the seedling stage (Supporting Information S9: Figure S9). *BHLH IVc* genes, *BHLH104, ILR3*, and *BHLH115*, were upregulated in the roots of the *pb104::b104^dPAA line #2* under +Fe compared to other lines (Figure 2D). Their target genes, such as *BHLH39, BHLH100, IMA1, BTS, BTSL1, BTSL2* and *PYE*, were also upregulated, indicating activation of the Fe deficiency response signalling, which includes a negative feedback loop via *BTS, BTSL1, BTSL2* and *PYE* genes. Likewise, the further downstream targets in roots, such as the Fe acquisition genes *IRT1*, *FRO2* and *FIT*, were up‐regulated (Figure 2D), consistent with the elevated Fe uptake and Fe content. Expression of negative regulators of the Fe deficiency response and of Fe sufficiency markers, such as *BHLH11* and *FER1*, was also increased, suggesting a proper response to elevated Fe (Figure 2D). In the shoot tissue, the Fe storage‐related gene *FER1* was strongly upregulated in *pb104::b104^dPAA line #2* compared to controls, regardless of Fe availability, in agreement with the elevated Fe content in this line (Figure 2D). Targeted expression profiling using reverse transcription quantitative PCR (RT‐qPCR) in seedling‐stage samples confirmed the expression patterns observed in the transcriptome analysis (Supporting Information S10: Figure S10).

To summarise, the misregulation of Fe response genes indicates that bHLH104 with deleted PAA disrupts normal Fe homeostasis by constitutively activating Fe acquisition while preventing proper feedback inhibition of Fe uptake during seedling development. This leads to excessive Fe acquisition and consequent toxicity.

### 2.3 b104^dPAA Causes Constitutive Fe Uptake and Transcriptional Misregulation at a Reproductive Growth Stage

Plants redistribute nutrients during reproduction to support flowering and seed development (Waters and Grusak 2008; Pottier et al. 2018). To assess whether b104^dPAA affects Fe homeostasis during later developmental stages, we analysed 30‐day‐old flowering *Arabidopsis* plants grown hydroponically, subjected at 27 days to a 3‐day ±Fe treatment (Figure 3A). However, the strong‐phenotype plants of *pb104::b104^dPAA line #2* were severely growth‐compromised in hydroponic culture and had to be excluded from this analysis. Instead, we focused on the weak‐phenotype line, *pb104::b104^dPAA line #3*. Old leaves, young leaves, and roots were collected for gene expression analysis, as described in Ngigi et al. (2025) (Figure 3C,D).

**Figure 3:**
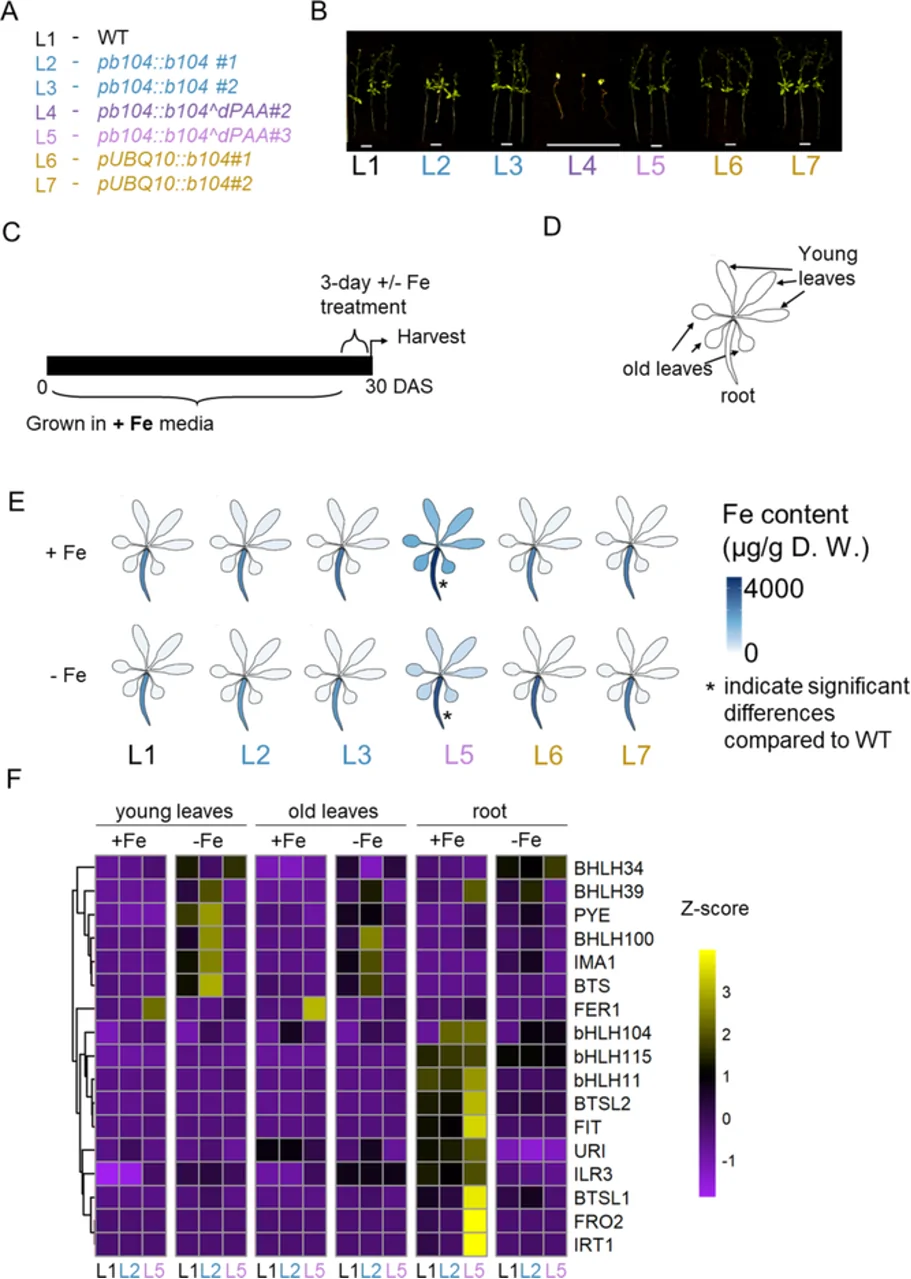
Elevated shoot iron accumulation and altered Fe deficiency responses in reproductive‐stage b104^dPAA transgenic lines. (A) Plant lines used in this experiment. (B) Representative images of 30‐day‐old *Arabidopsis* plants grown in a hydroponic system (scale bar = 5 cm). Among grown plant lines, *pb104::b104^dPAA* line #2 (L4) grew poorly and was not used in ICP‐MS and gene expression analysis. (C) Experimental design for the dataset shown. *Arabidopsis* plants were grown hydroponically under iron‐sufficient (+Fe) conditions for 27 days, followed by a 3‐day treatment with either +Fe or −Fe media. (D) Schematic representation of tissue separation used for both ICP‐MS and RT‐qPCR analysis. Young and old leaves were separated from the roots to examine tissue‐specific iron accumulation and gene expression. The same tissue layout is used to represent heatmaps in the ICP‐MS dataset. Leaves were grouped according to their morphological characteristics as described in Swartz et al. (2023) and Ngigi et al. (2025). The leaf arrangement in the schematic follows the heatmap layout used in (E) and (F) and does not indicate the position of the leaves on the plant. (E) Iron content in young leaves, old leaves, and roots under +Fe and −Fe conditions, measured by ICP‐MS. Data represent mean values from three biological replicates (*n* = 3). Heatmap intensity reflects iron concentration (µg/g dry weight). Colour shading was generated using the R package ggplantmap, with deeper blue indicating higher Fe content. Asterisks indicate genotypes that differ significantly from WT within the same tissue and treatment condition (*p* < 0.05), as determined by two‐way ANOVA and Tukey's post hoc test (*n* = 3 for each group). (F) Clustered heatmaps showing the expression profiles (RNA sequencing) of selected Fe homeostasis genes across genotypes, tissues (root, old leaves and young leaves) and treatments (+Fe and −Fe). Heatmaps were generated in RStudio version 4.4.1 using *Z*‐scores of normalised gene expression values calculated across all samples (*n* = 3 for each group). Purple indicates low expression, and yellow indicates high expression. Because *Z*‐scores are calculated across all samples to convey relative expression patterns, absolute expression magnitudes, including differences between root and leaves and between treatments, are not directly comparable within this representation. [Color figure can be viewed at wileyonlinelibrary.com]

Elemental profiling showed that the *pb104::b104^dPAA line #3* accumulated more Fe than control lines at the late developmental stage (Figure 3E). Under +Fe, roots of *pb104::b104^dPAA line #*3 had increased Mn and Zn levels compared to WT (Supporting Information S11: Figure S11). Cu levels were similar to WT.

In transcriptome analysis, the *pb104::b104^dPAA line #*3 plants had the highest DEG counts in young leaves (3877 DEGs) and old leaves (1895 DEGs) under +Fe conditions compared with controls, while root tissue exhibited minimal differential expression (71 DEGs) (Supporting Information S12: Figure S12A). GO term enrichment analysis revealed that genes associated with immune response were significantly upregulated in both old and young leaves of the *pb104::b104^dPAA line #*3 plants under +Fe conditions. In contrast, 3‐day −Fe treatment resulted in young leaves in the upregulation of terms related to Fe homeostasis (Supporting Information S12: Figure S12B). This indicates a tissue‐specific transcriptional response to Fe availability at the reproductive stage.

Specifically, expression analysis showed that in roots of *pb104::b104^dPAA line #3*, genes were upregulated under + Fe as reported above for *line #2* at the seedling stage, namely upregulation of *BHLH IVc* genes, Fe deficiency response, Fe sufficiency marker and negative *regulator* genes (Figure 3F). Fe deficiency response genes such as *BTS*, *IMA1*, *BHLH100*, and *PYE* were upregulated under −Fe in young leaves of *pb104::b104 #1* compared to *pb104::b104^dPAA* line #3 (Figure 3F). *FER1* was upregulated in young and old leaves of *pb104::b104^dPAA* line #3 compared to *pb104::b104* #1 and WT under +Fe. Targeted expression profiling by RT‐qPCR confirmed again the transcriptome data (Supporting Information S13: Figure S13). Hence, even though at the seedling stage, *pb104::b104^dPAA* line #3 did not display iron accumulation phenotypes, this was clearly the case at the flowering stage, indicating that proper bHLH104 function may be more important at later than early growth stages.

Finally, as *pb104::b104^dPAA* line #2 did not grow in +Fe hydroponic conditions, we modified the hydroponic growth system and changed to an Fe resupply assay. Seedlings were transferred at an early stage to −Fe (instead of the usual +Fe) medium for 27 days, followed by a 3‐day ±Fe resupply. While WT plants remained chlorotic and accumulated Fe mostly in the roots following the Fe resupply, *pb104::b104^dPAA* line #2 showed substantial Fe accumulation in roots and leaves despite persistent leaf chlorosis, suggesting high root‐to‐shoot Fe translocation. Notably, the *pb104::b104^dPAA* line #2 accumulated significantly less Mn, Zn, Cu compared to control plants in old and young leaves (Supporting Information S14: Figure S14). This shows that at the reproductive stage, there is a particularly elevated Fe uptake and translocation towards shoots in the *pb104::b104^dPAA line #*2 compared to the WT, while that of Mn, Zn and Cu is suppressed.

Since the 30‐day‐old *pb104::b104^dPAA line #*3 plants accumulated not only Fe but also Mn and Zn under +Fe in roots, we examined the possibility that this might have been caused by altered expression of metal transporter genes. We found elevated expression of metal transporter genes, including *NRAMP1*, *COPT2*, *MTP8*, and *ZIP* family members, in *pb104::b104^dPAA* roots under both +Fe and −Fe at the seedling stage, and under +Fe at the reproductive stage, consistent with the observed metal accumulation patterns (Supporting Information S15: Figure S15).

To summarise, *pb104::b104^dPAA* lines accumulated Fe throughout the tested reproductive stage. Metal accumulation and associated phenotypes due to b104^dPAA expression remained consistent from seedling to advanced developmental stages.

### 2.4 The PAA Sequence Is Required for 26S Proteasome‐Mediated Degradation of bHLH104

The severity of phenotypes in *pb104::b104^dPAA* lines was not explained by differences in transgene transcript levels (Supporting Information S10: Figure S10C). Additionally, *pUBQ10::b104* lines showed no significant phenotype compared to WT apart from shorter roots despite elevated *bHLH104* transgene expression (Supporting Information S6: Figure S6, Supporting Information S10: Figure S10C). Therefore, we suspected that variation in bHLH104 protein accumulation underlies the observed phenotypic differences.

We thus examined in vivo accumulation of mTurquoise2‐tagged bHLH104 and b104^dPAA protein, expressed under either the *UBQ10* or *BHLH104* promoter (Figure 4A,B). Immunoblotting of root tissue proteins detected mTurquoise2‐b104^dPAA protein in *pb104::b104^dPAA* lines #1 and #2 (Figure 4B, Supporting Information S16: Figure S16). In contrast, the mTurquoise2‐tagged protein was barely detectable in *pb104::b104^dPAA* lines #3 and #4, *pb104::b104* and *pUBQ10::b104* lines. Hence, mTurquoise2‐b104^dPAA protein levels coincided with the strength of Fe accumulation phenotypes, while the WT mTurquoise2‐b104 was not detected.

**Figure 4:**
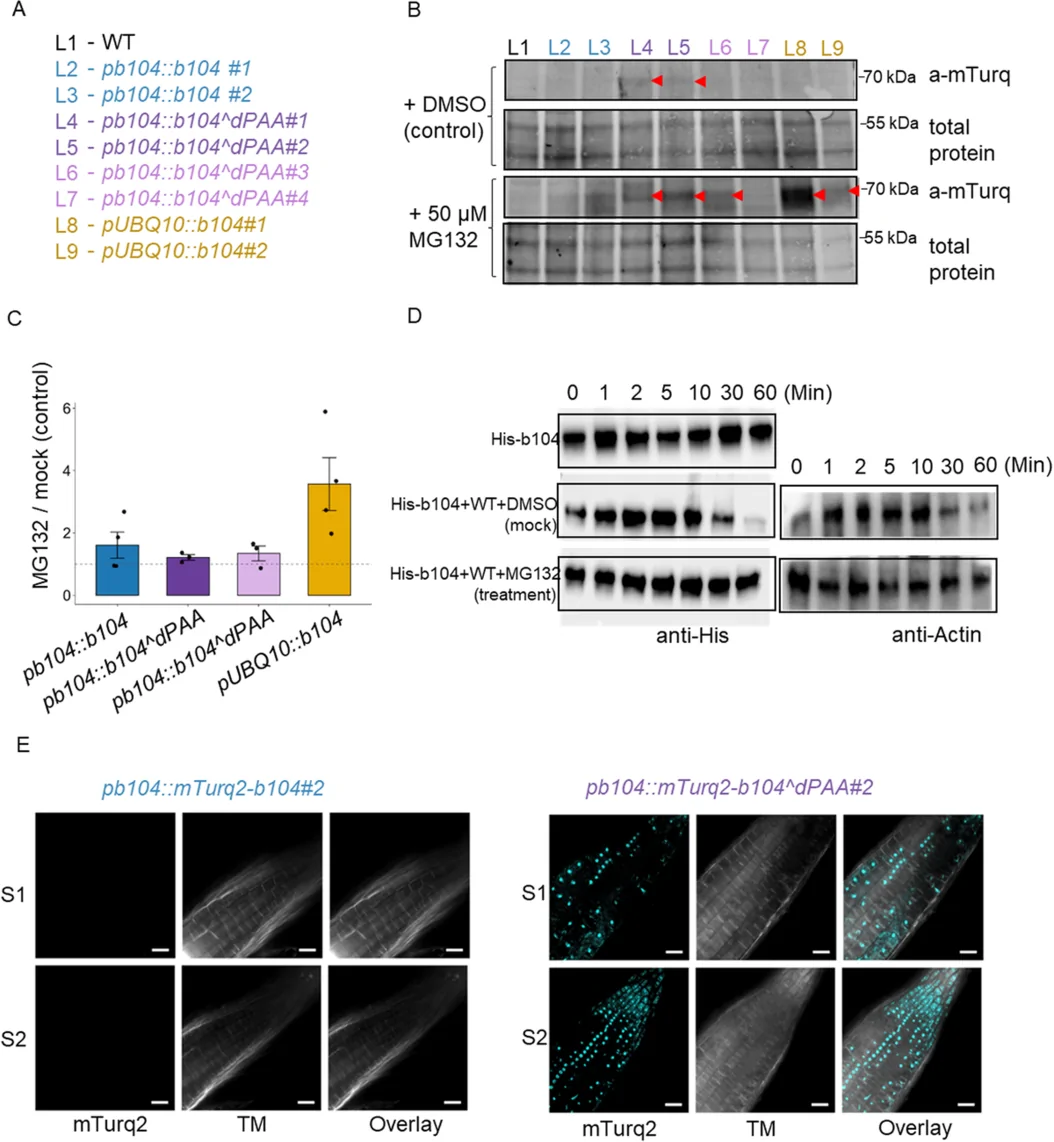
The PAA sequence mediates bHLH104 protein stability and affects its abundance in *Arabidopsis*. (A) Genotypes and transgenic lines used in this experiment. (B) Immunoblot analysis of mTurquoise2‐tagged bHLH104 and b104^dPAA in different transgenic lines treated with either DMSO (control) or 50 μM MG132. Total protein and anti‐mTurquoise2 blots are shown. Samples are labelled from #L1 to #L9. The transgenic lines exhibiting increased protein abundance correspond to those that displayed altered growth phenotypes in Figure 1, suggesting a link between protein stability and the observed developmental responses. Red arrows indicate the expected band position for mTurquoise2‐bHLH104 fusion protein. Additional blots for replicate studies are provided in Supporting Information S16: Figure S16. Original uncropped blot is shown in Supporting Information S23: Figure S23. (C) Relative protein levels of mTurquoise‐tagged bHLH104 were determined by Western blot. Band intensities were normalised to total protein, and fold‐change was calculated as the ratio of MG132‐treated to DMSO‐treated samples. Bars represent mean ± SEM; individual data points are shown. (D) Cell‐free degradation assay showing in vitro degradation of His‐tagged bHLH104 protein when incubated with protein extracts from 10‐day‐old Col‐0 (WT) *Arabidopsis thaliana* seedlings. His‐bHLH104 was expressed in *Escherichia coli* and remained stable on its own, but degraded upon incubation with the plant extracts, indicating proteasome‐mediated degradation. Total protein from whole seedlings was extracted using standard protein extraction procedures. Samples were collected at the indicated time points and analysed by immunoblotting with anti‐His and anti‐Actin antibodies. MG132 treatment stabilises bHLH104, indicating proteasome‐dependent degradation. Relative His‐bHLH104 levels were quantified and normalised to total lane protein, and Actin band intensities at the corresponding time points are shown separately (Supporting Information S17: Figure S17). (E) Confocal microscopy of mTurquoise2 fluorescence in roots of transgenic *Arabidopsis* seedlings expressing full‐length bHLH104 (left) or b104^dPAA (right). Images show two representative seedlings (S1 and S2) per genotype. *A. thaliana* seedlings were at the germination stage, 5 days old, corresponding to 2 days after stratification, and grown on +Fe medium. TM, transmitted light; Overlay, merged mTurq2 and TM channels. Scale bar = 25 µm; excitation at 458 nm; emission collected from 493 to 613 nm. [Color figure can be viewed at wileyonlinelibrary.com]

To check whether the low abundance of mTurquoise2‐bHLH104 is due to proteasomal degradation, we treated samples with proteasome inhibitor MG132. MG132 treatment stabilised full‐length mTurquoise2‐bHLH104 in *pUBQ10::b104* lines by more than two‐fold. However, MG132 treatment did not produce a detectable mTurquoise2‐bHLH104 signal in the native promoter lines (Figure 4B,C). To confirm that bHLH104 is subject to proteasomal degradation, a cell‐free degradation assay was performed. When recombinant His‐bHLH104 protein, purified from *Escherichia coli*, was incubated with whole protein extracts from WT seedlings, it was rapidly degraded. This degradation was inhibited by the proteasome inhibitor MG132, supporting the involvement of the 26S proteasome (Figure 4D).

Confocal imaging of root tips further confirmed the increased abundance of mTurquoise2‐b104^dPAA, as indicated by fluorescence signals in nuclei of root tip cells in *pb104::b104^dPAA* lines, whereas fluorescence was undetectable in lines expressing full‐length mTurquoise2‐bHLH104 (Figure 4E).

Altogether, these findings show that the PAA sequence is required for degradation of bHLH104 by the 26S proteasome. Removing this sequence prevents normal turnover of the protein.

### 2.5 The C‐Terminal PAA Sequence of bHLH104 Mediates Interaction With BTS E3 Ligases and Enables Ubiquitination

To understand how the PAA sequence contributes to bHLH104 degradation, we examined its role in mediating interaction with BTS.

AlphaFold3 modelling (Abramson et al. 2024) predicted an interface with high predicted Local Distance Difference Test (pLDDT) scores, indicating high confidence in the predicted structure. Electrostatic surface analysis (Pettersen et al. 2004) showed that this interface is charge‐based (Figure 5A,B). These models suggest that the PAA sequence forms part of the predicted interaction surface with BTS. To examine the contribution of the individual residues within this sequence, we substituted each position of the PAA sequence in silico in the AlphaFold3 models and quantified the effect on the predicted interface as the change in interface predicted aligned error (ΔiPAE) relative to the WT sequence, where higher values indicate a weaker predicted interaction (Figure 5C). Substitution of the terminal alanine weakened the predicted interface, consistent with its reported importance for BTS binding (Sharma and Yeh 2020; Li et al. 2021). Substitution of the proline weakened the interface to a comparable extent. At the central position, the effect depended on the residue introduced: substitution of alanine by valine, which generates the PVA sequence found in other bHLH IVc proteins, had no significant effect, whereas substitution by glycine weakened the predicted interface. These predictions indicate that recognition involves the PAA sequence as a unit, with both the proline and the terminal alanine contributing to the predicted interface, rather than the terminal alanine alone. The substitutions were modelled and not tested experimentally, and the contribution of each position therefore requires confirmation.

**Figure 5:**
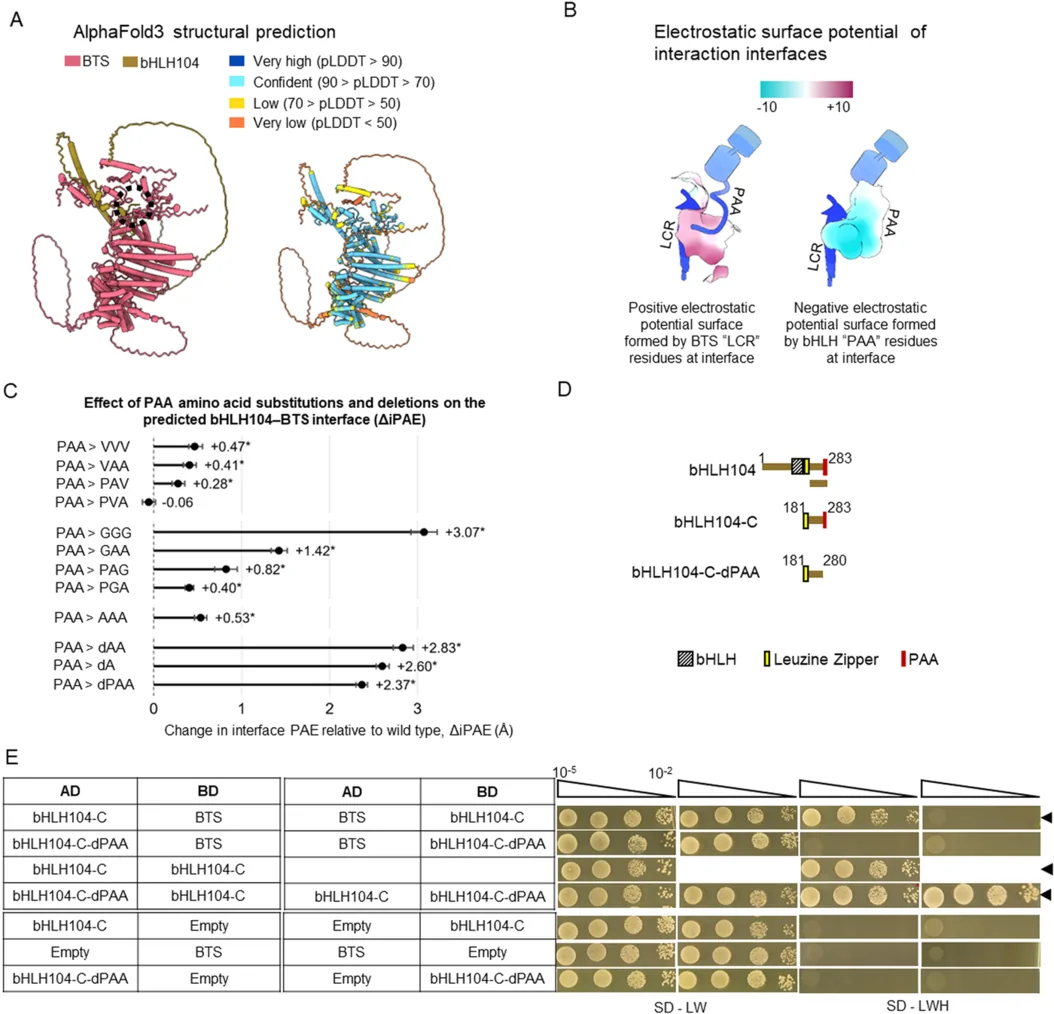
The PAA sequence is required for bHLH104 interaction with BTS. (A) Structural prediction of the bHLH104–BTS complex using AlphaFold3 (Abramson et al. 2024). Left: Chain‐coloured model showing BTS (rose colour) and bHLH104 (bronze colour). Right: Per‐residue confidence model with pLDDT scores colour‐coded as very high (pLDDT > 90), confident (90 > pLDDT > 70), low (70 > pLDDT > 50), and very low (pLDDT < 50). A dashed circle marks the interaction interface formed by BTS and bHLH104. (B) Electrostatic surface potential modelling (Pettersen et al. 2004) reveals a negatively charged region near the proline–alanine–alanine (PAA) sequence in bHLH104 that complements a positively charged surface in the leucine–cysteine–arginine (LCR) domain of BTS. The electrostatic scale ranges from −10 (purple) to +10 (cyan) kT/e. (C) In silico substitution analysis of the C‐te rminal PAA sequence in the predicted bHLH104‐BTS interface. Each of the three C‐terminal residues was substituted individually by valine, glycine or alanine, and C‐terminal deletion variants lacking one, two or all three residues were modelled with AlphaFold3. The effect of each variant on the predicted interface is shown as the change in interface predicted aligned error (ΔiPAE, in Å) relative to the wild‐type PAA sequence, where higher values indicate a weaker predicted interaction. To compare all variants against the same site, a fixed set of BTS interface residues defined from the wild‐type complex was applied to every construct. Labels read as ‘PAA > variant’; dPAA denotes deletion of PAA, dA deletion of the last alanine, and dAA deletion of the last two alanines. Points show the mean ΔiPAE across five models per construct; error bars indicate bootstrap confidence intervals. Asterisks denote variants with a significant effect on the predicted interface; variants without an asterisk, such as PAA > PVA, are not significant. (D) Schematic representation of full‐length bHLH104, bHLH104‐C and bHLH104‐C^dPAA used for Y2H and UbiGate assays. C‐terminal parts lack the N‐terminus and the DNA‐binding basic Helix Loop Helix (bHLH) domain. LZ is the Leuzine zipper domain. Numbers indicate amino acid positions. (E) Yeast two‐hybrid assays testing protein–protein interactions between bHLH104‐C^dPAA and BTS. bHLH104‐C and BTS heterodimerisation and bHLH104‐C homodimerisation serve as positive controls. Note that C‐terminal bHLH104 fragments were used since full‐length bHLH104 shows autoactivation in Y2H (Lichtblau et al. 2022). The data in Figure 5E and Supporting Information S18: Figure S18 derive from a single yeast two‐hybrid experiment in which all combinations were transformed, plated and imaged in parallel. The empty vector controls bHLH104‐C + Empty, Empty + bHLH104‐C, b104‐C^dPAA + Empty and Empty + b104‐C^dPAA are therefore shown as the same images in both figures. Black arrows indicate positive interaction due to colony growth in the selective condition. [Color figure can be viewed at wileyonlinelibrary.com]

To further study protein interaction in the presence and absence of PAA experimentally, we made use of established protein expression systems. Due to the difficulties of detecting bHLH104 and BTS in plant cell assays, we used the Y2H assay (Lichtblau et al. 2022). BTS interacts non‐reciprocally with the C‐terminal part of bHLH104 (bHLH104‐C) in yeast, and bHLH104‐C does not auto‐activate in the Y2H system (Figure 5E), as previously shown (Lichtblau et al. 2022). We now tested whether interaction is different with bHLH104‐C^dPAA. Indeed, deletion of the PAA sequence abolished the interaction with BTS (Figure 5E). bHLH104 is known to homodimerise (Zhang et al. 2015; Li et al. 2016; Lichtblau et al. 2022). bHLH104‐C^dPAA still dimerised with bHLH104‐C, confirming that bHLH104‐C^dPAA is present and properly folded in yeast cells. A similar loss of interaction ability was found between bHLH104‐C^dPAA and BTSL1 and BTSL2 (Supporting Information S18: Figure S18), indicating that the PAA sequence is broadly required for recognition by BTS/BTSL E3 ligases.

To test whether bHLH104 is ubiquitinated by BTS with the requirement of the PAA sequence, we established the bacterial UbiGate system (Kowarschik et al. 2018; Ortmann et al. 2023) to reconstitute a BTS‐dependent ubiquitin‐conjugation cascade for bHLH104 (Figure 6A). The UbiGate system co‐expresses all ubiquitination and target components within living bacterial cells, providing a cellular context for a direct ubiquitination reaction while enabling assignment of E3‐substrate specificity through distinct epitope tags on each component (Figure 6B, Supporting Information S20: Figure S20). Since full‐length BTS was not stably expressed in *E. coli*, we used a truncated version (BTS‐HC) lacking the first two hemerythrin domains.

**Figure 6:**
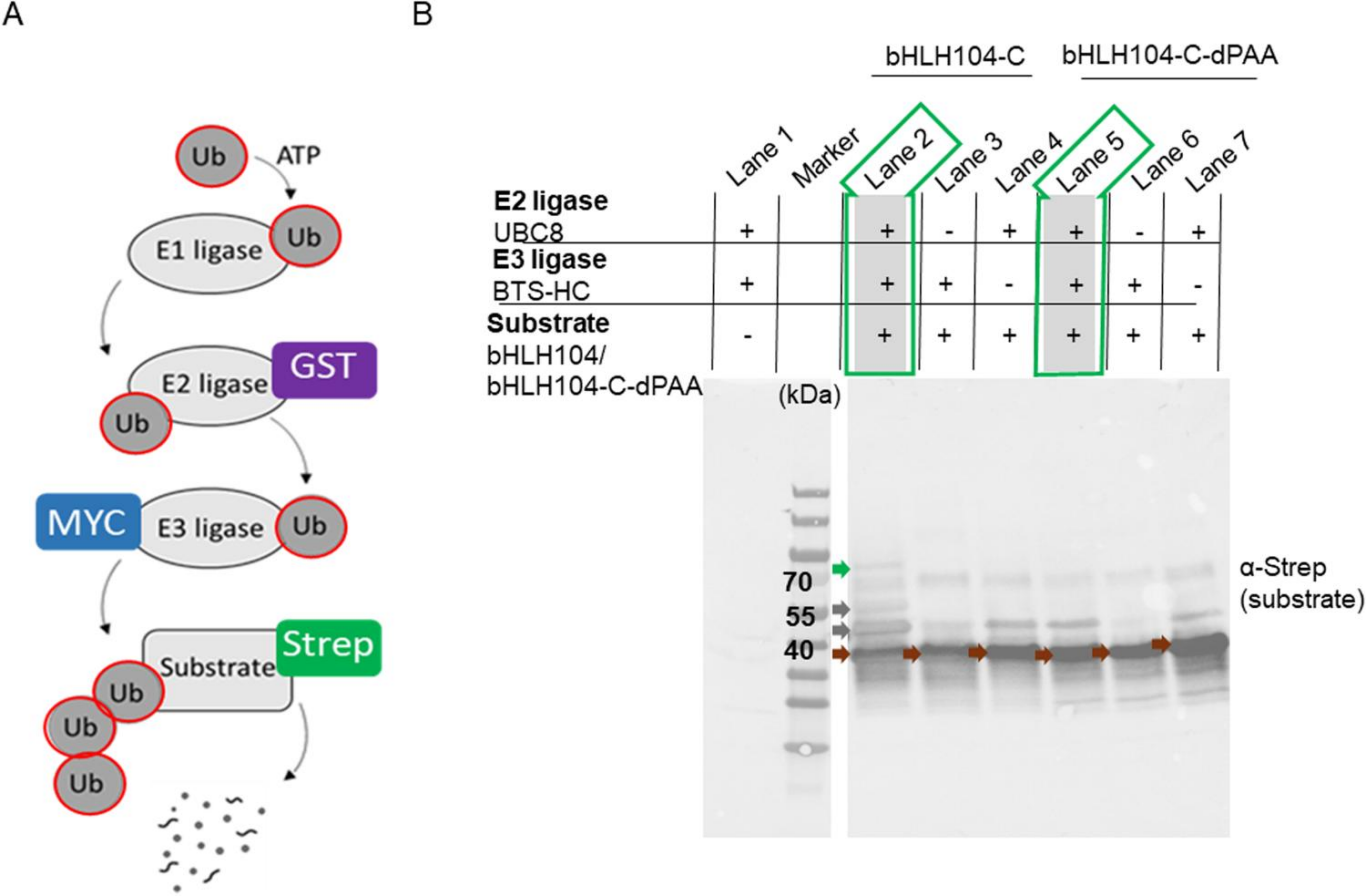
The PAA sequence leads to a specific bHLH104‐C band in the UbiGate system testing for BTS‐mediated ubiquitination of bHLH104 in *Escherichia coli* cells. (A) Schematic overview of the ubiquitin/26S proteasome pathway. Each component was expressed in *E. coli* using the UbiGate system (Kowarschik et al. 2018). Recombinant bHLH104‐C or bHLH104‐C^dPAA proteins (Strep‐tagged) were used as substrates. BTS‐HC served as the E3 ligase (Myc‐tagged), in combination with UBC8 as E2 ligase (GST‐tagged), an E1 activating enzyme, and ubiquitin (His‐tagged). (B) Anti‐Strep immunoblot detecting the Strep‐tagged substrate. The key comparison is between lane 2 and lane 5 (green boxes), in which E2, E3 and substrate were all present, and which differ only in the substrate used: bHLH104‐C in lane 2 and bHLH104‐C^dPAA in lane 5. A band at ~70 kDa (green arrow) appears with bHLH104‐C (lane 2) but not with bHLH104‐C^dPAA (lane 5). This band lies ~30 kDa above the unmodified substrate, consistent with the addition of three to four ubiquitin moieties (~8.6 kDa each) and with polyubiquitination of bHLH104‐C (Kowarschik et al. 2018). The remaining lanes are controls. Lane 1 contains E2 and E3 without substrate. Lanes 3 and 6 lack E2 ligase, and lanes 4 and 7 lack E3 ligase, for bHLH104‐C and bHLH104‐C^dPAA, respectively. The ~70 kDa band is absent from all of these lanes. The anti‐Strep antibody detects the unmodified substrate at ~40 kDa (brown arrows) in all lanes containing substrate. Additional higher‐molecular‐weight bands that also occur without the E3 ligase (grey arrows) are not specific to BTS‐HC activity. The assay was performed three times with the same result for the ~70 kDa band (additional replicates in Supporting Information S19: Figure S19). [Color figure can be viewed at wileyonlinelibrary.com]

Substrate ubiquitination was assessed on the anti‐Strep blot, since the anti‐Strep antibody detects only the Strep‐tagged substrate (bHLH104‐C or bHLH104‐C^dPAA). The relevant comparison is between lane 2 and lane 5 of Figure 6B, in which E2, E3 and substrate were all present and which differ only in the substrate used. With bHLH104‐C (lane 2), an additional band appeared at ~70 kDa (green arrow), corresponding to a shift of ~30 kDa above the unmodified protein, consistent with the addition of three to four ubiquitin moieties and with polyubiquitination of bHLH104‐C (Kowarschik et al. 2018); this meets the minimum chain length of four ubiquitins required for efficient 26S proteasome recognition (Thrower et al. 2000). This band was absent with bHLH104‐C^dPAA (lane 5). It was also absent from the control lanes, in which E2 ligase was omitted (lanes 3 and 6), E3 ligase was omitted (lanes 4 and 7), or the substrate was omitted (lane 1). In all lanes containing substrate, the anti‐Strep antibody detected the unmodified protein at the expected size (~40 kDa; brown arrows). The ~70 kDa band was reproducible across three independent experiments (Supporting Information S19: Figure S19). Some higher‐molecular‐weight bands occurred independently of the E3 ligase (grey arrows) and are therefore not specific to BTS‐HC activity.

The anti‐His‐Ub blot (Supporting Information S20: Figure S20) detects total ubiquitin conjugates in the reaction, including E2 and E3 (BTS) autoubiquitination, and is therefore not a substrate‐specific readout. It documents that the ubiquitination cascade was active, and we did not use band densities on this blot to assess substrate ubiquitination. BTS autoubiquitination, reported previously (Kobayashi et al. 2013; Selote et al. 2015), served as a positive control for the reaction (anti‐myc blot; Supporting Information S20: Figure S20). Consistent with the published role of bHLH115 as a BTS target (Selote et al. 2015; Xing et al. 2021), bHLH115 also showed evidence of ubiquitination by BTS‐HC in this assay, supporting the suitability of the system (Supporting Information S21: Figure S21).

These data indicate that bHLH104 can act as a substrate of BTS in a cellular environment and that the PAA sequence is required for its ubiquitination. The specificity is supported by the bHLH104‐C^dPAA control, which differs from bHLH104‐C by only three C‐terminal residues yet does not produce the ~70 kDa band.

## 3 Discussion

This study demonstrates the functional relevance of the terminal PAA sequence of bHLH104 through a PAA deletion variant. Our data indicate that loss of PAA in bHLH104 prevents its binding to the BTS E3 ligase, stabilising b104^dPAA and constitutively activating the Fe deficiency response, which leads to accumulation of Fe and other metals. The extent of these effects differs between transgenic lines: some lines show oxidative stress and growth defects, whereas others accumulate Fe over their life span without compromised growth. Hence, manipulation of the terminal PAA sequence can lead to accumulation of Fe and other micronutrients in leaves, provided that bHLH104 protein levels remain below those that compromise growth.

### 3.1 bHLH104 Abundance Is Controlled via the Proteasome

bHLH104 is regulated at the protein level, in agreement with reports for other bHLH IVc proteins (Zhang et al. 2015; Li et al. 2016; Liang et al. 2017; Selote et al. 2015). Two findings indicate that this regulation is post‐translational rather than transcriptional: bHLH104 protein abundance does not follow *BHLH104* transcript abundance in the transgenic lines, and the full‐length bHLH104 protein is detected only after proteasome inhibition. bHLH104 turnover was more rapid in the assay in this study than in the cell‐free assay of Selote et al. (2015), in which the protein remained stable for 8 h; this difference may reflect the properties of the recombinant protein produced in the two expression systems. Our UbiGate data extend this model to bHLH104 specifically, indicating that BTS can ubiquitinate the protein in a PAA‐dependent manner. BTSL1 and BTSL2 have recently been shown to target bHLH105 and bHLH115 for degradation in roots (Zhao et al. 2026), consistent with redundant activity of BTS‐type E3 ligases in roots. Degradation of bHLH104 may also proceed independently of the PAA sequence. Such degradation could involve additional E3 ligase systems, as reported for the apple orthologue MdbHLH104 (targeted by MdBT2‐MdCUL3 independently of HRZ/BTS; Zhao et al. 2016), or selective autophagy (Wang et al. 2021; Raffeiner et al. 2023). Despite elevated mTurq2‐bHLH104 transcript levels in shoots, *pUBQ10::b104* lines showed no phenotype, in contrast to previously reported *p35S::bHLH104‐GFP* lines that displayed enhanced Fe deficiency tolerance, increased ferric‐chelate reductase activity, and Fe overaccumulation (Zhang et al. 2015). Several factors may account for this discrepancy. The 35S promoter typically drives stronger expression than the *UBQ10* promoter, potentially saturating BTS‐mediated degradation capacity. Additionally, the C‐terminal GFP tag in *35S::bHLH104‐GFP* lines may affect BTS accessibility to the adjacent PAA sequence, whereas an N‐terminal mTurquoise2 tag leaves the C‐terminus unobstructed. By contrast, the b104^dPAA lines showed upregulation of *BHLH39* and *BHLH100* in roots under both +Fe and −Fe conditions, indicating that the functional output of bHLH104 depends on protein abundance rather than on transcript abundance. Whether the observed upregulation of *BHLH IVc* genes in the b104^dPAA lines reflects direct transcriptional autoregulation in a positive feedback loop or indirect effects through altered Fe status remains to be determined. Future studies can investigate additional post‐translational modifications and identify environmental and physiological conditions that promote bHLH104 stabilisation.

### 3.2 b104^dPAA Stimulates Fe and Metal Ion Accumulation and Potential for Biofortification

The accumulation of metals other than Fe can be explained by persistent activation of a broad array of metal transporters in root cells, such as the Fe, Zn and Mn transporter IRT1, the Fe and Mn transporter NRAMP1, the Cu transporter COPT2 and the Mn transporter MTP8 (Connolly et al. 2002; Vert et al. 2002; Cailliatte et al. 2010; Eroglu et al. 2016). The genes encoding these transporters are under the control of the FIT transcription factor (Schwarz and Bauer 2020), and the FIT pathway was found active downstream of bHLH104 in our study. Since b104^dPAA plants accumulate excess metals, including Zn, this could further amplify the Fe deficiency signal. This is consistent with reports that heavy metals such as Zn can induce Fe deficiency responses at the level of Fe sensing rather than by directly inhibiting Fe transport (Lešková et al. 2017), and with the demonstration that Zn or Fe binding to multiple domains of HRZ E3 ligases modulates their stability and function in response to Fe nutritional status (Shinkawa et al. 2025). Thus, the accumulated metals in *pb104::b104^dPAA* plants may reinforce Fe deficiency signalling, which could contribute to a positive feedback loop sustaining constitutive Fe uptake. Similar phenotypes where Fe accumulation disrupts plant growth have been reported in *BTS* loss‐of‐function mutants and in overexpression lines of *BHLH39* or *BHLH105* (Zhang et al. 2015; Li et al. 2016; Hindt et al. 2017; Naranjo‐Arcos et al. 2017). A still unresolved question is why the known negative post‐translational regulation mechanisms, e.g. IRT1 ubiquitination and degradation under low Fe and excessive Zn and Mn, are not in place here (Dubeaux et al. 2018; Abuzeineh et al. 2022). One possibility is that this regulation requires Zn and Mn to be supplied in the rhizosphere rather than inside the root. In our experimental settings, excessive Zn and Mn were not supplied, and Zn and Mn accumulated due to a plant internal imbalance.

Notably, the *pb104::b104^dPAA line #3*, which showed negligible b104^dPAA protein in seedling roots, grew normally during early development but accumulated Fe in leaves at the reproductive stage. This indicates that a low level of b104^dPAA activity can increase Fe content without causing severe growth defects, and points to a threshold below which Fe accumulation is enhanced without compromising growth. Defining this threshold and separating the contribution of b104^dPAA from that of endogenous bHLH104, which is also present in these lines, will be required before the system can be used for Fe biofortification. More broadly, the findings integrate with emerging work on BTS/BTSL hemerythrin‐based Fe/oxygen sensing (Selote et al. 2015; Pullin et al. 2025) and clarify how post‐translational control translates Fe status into transcriptional outputs.

### 3.3 Pleiotropic and Tissue‐Specific Effects of b104^dPAA

The phenotypes observed in *pb104::b104^dPAA* plants extend beyond simple Fe accumulation, suggesting that constitutive Fe deficiency signalling has broader physiological consequences. Several phenotypes observed in these lines may reflect activation of multiple stress regulatory pathways. First, the delayed flowering phenotype may result from −Fe response signalling interfering with flowering time control through epigenetic, circadian, and hormone pathways (Cheng et al. 2004; Salomé et al. 2012; Chen et al. 2013; Hong et al. 2013; Wild et al. 2016; Dutta et al. 2017; Maruoka et al. 2022). Second, the enrichment of immune and salicylic acid signalling terms in *pb104::b104^dPAA line #3* leaves is consistent with crosstalk between Fe deficiency signalling and immunity. Fe deficiency treatment elevates salicylic acid levels and induces salicylic acid‐responsive genes (Shen et al. 2016), and bHLH105 integrates Fe homeostasis with plant immune responses against pathogens (Rampey et al. 2006; Aparicio and Pallás 2017; Samira et al. 2018). We therefore distinguish the direct downstream effects of stabilised bHLH104, seen in the coordinated misregulation of the Fe‐acquisition and homeostasis regulon, from the broader transcriptional changes, including the immune and salicylic‐acid signatures and the hypoxia and oxidative‐stress terms, which we interpret as indirect consequences of the altered Fe and metal status rather than direct bHLH104 targets.

Expression of b104^dPAA also had distinct tissue‐specific consequences. In leaves of mature *pb104::b104^dPAA* lines, internal Fe allocation genes such as *BTS, PYE*, *IMA1, BHLH100* and *BHLH39* were not upregulated under −Fe, suggesting that elevated local leaf Fe suppresses the Fe deficiency response downstream of bHLH104, consistent with evidence that leaf Fe responses are determined mainly by local Fe status rather than root‐derived systemic signals (Nguyen et al. 2022; Ngigi et al. 2025). In contrast, the −Fe acquisition genes *FIT, IRT1* and *FRO2* were upregulated in roots. In WT plants, bHLH104 is expected to be stabilised only under Fe deficiency, enabling transient activation of uptake genes, whereas loss of PAA prevents this regulation, so that Fe uptake continues regardless of Fe status. Consistent with this, we observed enhanced Fe mobilisation to young leaves in b104^dPAA line #2 after 3 days of Fe resupply, while WT plants retained most Fe in roots. This may be partly explained by elevated *IRT1* mRNA expression, as *IRT1* protein is required for effective root‐to‐shoot Fe partitioning independent of its transport activity (Quintana et al. 2022). Thus, bHLH104 protein stability acts as a control point linking Fe sensing to the root acquisition response, whereas leaves respond mainly to local Fe. Grafting experiments would help determine whether these effects are organ‐autonomous or involve systemic signalling. *BHLH IVc* genes, including bHLH104, are expressed in the root tip (Li et al. 2016; Liang et al. 2017; Gao et al. 2024), and the accumulation of stabilised b104^dPAA in this region, where the native protein is not detected, may reflect loss of PAA‐dependent turnover of a protein that is otherwise maintained at low abundance by BTS/BTSL activity.

### 3.4 The PAA Sequence as a Recognition Motif in bHLH IVc Protein Regulation

Our findings indicate that recognition of bHLH104 by BTS involves the C‐terminal PAA as a unit rather than the terminal alanine alone. This is compatible with the earlier reports. Substitution of the terminal alanine is sufficient to abolish the interaction (Li et al. 2021; Sharma and Yeh 2020; Zhao et al. 2026), but that result establishes the terminal residue as necessary and does not exclude a contribution from the adjacent positions, which was not tested. Substitution of the proline weakened the predicted interface to a comparable extent, placing it within the same recognition determinant. At the central position, a side chain appears to be required rather than a particular residue. This degeneracy is consistent with the occurrence of PVA in the other bHLH IVc proteins, which are likewise BTS targets, and with the presence of PAA in the IMA proteins, where the same sequence is read by BTS/L. These conclusions rest on structure predictions, and the contribution of the individual positions remains to be tested in binding assays. Our data establish the PAA sequence as necessary for bHLH104 turnover; its sufficiency is untested. Appending PAA to an unrelated transcription factor would address this and would show whether the motif can be used to place other proteins under Fe‐dependent stability control.

### 3.5 Concluding Remarks and Perspectives

The functional output of bHLH104 depends on its protein abundance rather than on its transcript abundance, and the present study identifies the three C‐terminal residues of the protein as a determinant of that abundance. In their absence, bHLH104 escapes BTS‐mediated turnover and accumulates, and the Fe deficiency response is activated under Fe‐sufficient conditions at both the seedling and the reproductive stage (Figure 7). Across independent transgenic lines, phenotype severity and Fe content correlated with the amount of stabilised protein, indicating that the effect of the deletion is graded with protein abundance rather than all‐or‐none. Fe acquisition in *A. thaliana* is therefore subject to post‐translational control through a sequence of three amino acids, in vegetative as well as in reproductive tissues.

**Figure 7:**
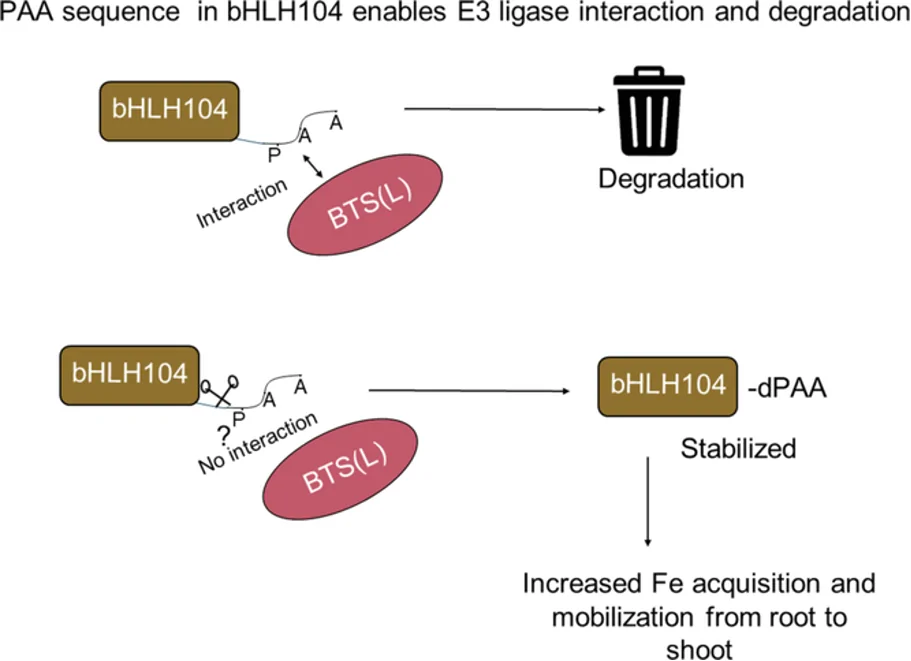
Working model of bHLH104 regulation through the C‐terminal PAA sequence and its impact on Fe homeostasis. The C‐terminal PAA sequence in bHLH104 mediates interaction with BTS‐type E3 ligases, targeting the protein for proteasomal degradation. Deletion of the PAA motif (b104^dPAA) disrupts this interaction, leading to protein stabilisation. The increased abundance of stabilised b104^dPAA enhances Fe acquisition responses in *Arabidopsis*. [Color figure can be viewed at wileyonlinelibrary.com]

In contrast to whole‐gene overexpression, precise editing of the terminal residues may offer a means to increase Fe content while minimising pleiotropic effects. As bHLH IVc proteins are conserved across plant species (Jiang et al. 2024), this could be exploited for Fe biofortification. The strong‐phenotype lines carry developmental costs, so application would require lines in which Fe accumulation is achieved without growth penalties. Because the PAA sequence is also present in IMA proteins, which positively regulate Fe uptake, such approaches would need to target bHLH IVc genes specifically.

## 4 Materials and Methods

### 4.1 Gene Accession Numbers

The following *A. thaliana* genes were the focus of this study: bHLH104 (AT4G14410), *BTS/BRUTUS* (AT3G18290), *BTSL1* (AT1G74770), and *BTSL2* (AT1G18910).

### 4.2 Plant Material and Growth Conditions

*A. thaliana* Columbia‐0 (Col‐0) was used as the WT in this study. The transgenic lines were generated using the floral dip method using *Agrobacterium tumefaciens* (strain GV3101 pMP90 pSoup) (Clough and Bent 1998). Constructs for generating transgenic lines were assembled using the GreenGate cloning system (Lampropoulos et al. 2013). The following modules were assembled into pGGZ003 destination vector: Promoter sequence (pUBQ10/pbHLH104 (1636‐bp sequence upstream of *BHLH104* gene)), an N‐terminal mTurquoise2 fluorescent tag, coding sequence (BHLH104*/BHLH104^dPAA*), bHLH104 terminator sequence (975 bp), and hygromycin selection marker. Primers used are listed in Supporting Information S24: Table S1.

For plate‐based experiments, plants were grown as described in Lingam et al. (2011) and Lichtblau et al. (2022) in modified half‐strength Hoagland medium. Fe was supplied as 50 µM Fe(III)‐EDTA for +Fe or omitted for −Fe conditions. The efficacy of Fe deficiency treatment was validated by confirming upregulation of canonical Fe deficiency marker genes (*IRT1, FRO2, bHLH39, bHLH100*) in WT plants (Supporting Information S22: Figure S22).

All plants for the phenotyping experiments were genotyped for the presence of the transgene. For hydroponic experiments, seedlings were initially grown on modified half‐strength Hoagland agar plates for 12 days before being transferred to a hydroponic setup as described in Ngigi et al. (2025). Young and old rosette leaves were distinguished and grouped based on their morphological characteristics, following Swartz et al. (2023) and Ngigi et al. (2025).

For soil‐based phenotyping experiments, seedlings were transferred to soil on the eighth day after germination. Phenotypic measurements were taken after 3 weeks of soil growth.

### 4.3 Phenotyping and Image Acquisition

Automated phenotyping was performed using the MicroScan system equipped with a PlantEye F600 sensor (Phenospex, Heerlen, The Netherlands) and controlled by the implemented software HortControl (Phena version 2.0, HortControl version 3.85) as previously described (Tripodi et al. 2024; Knopf and Bauer 2025). The PlantEye F600 acquires a point cloud of the plant by laser triangulation, identifies contiguous surfaces within the point cloud and fits triangles between individual points, from which morphological parameters are derived (Manavalan et al. 2021). The parameter reported here as 3D leaf area is the summed area of the fitted mesh and therefore represents the leaf surface area of the three‐dimensional reconstruction. It differs from the projected leaf area obtained by top‐down two‐dimensional imaging in that it follows the non‐planar surface of the leaf and accounts for leaf curvature, leaf angle and hyponasty. Spectral parameters included NDVI, calculated as (near‐infrared reflectance [NIR] − red reflectance [RED])/(NIR + RED).

Manual morphological phenotyping, like flowering time, was performed as described in Knopf and Bauer (2025).

Fe reductase activity and root length were measured using protocols described in Gratz et al. (2019). Each assay included at least three biological replicates.

Seed number was estimated based on seed weight. For each biological replicate, 100 seeds were manually counted and weighed to determine the mean weight per 100 seeds. The total seed number was calculated as (total seed weight/weight of 100 seeds) × 100. Three biological replicates were analysed for each genotype.

### 4.4 Plant Protein Extraction and Immunoblotting

Total proteins were extracted from roots of *A. thaliana* seedlings grown in modified half‐strength Hoagland media plates. Proteins were solubilised in 2× SDG buffer (125 mM Tris‐HCl, pH 6.8, 5% SDS, 4% DTT, 20% glycerol, 0.02% bromophenol blue) at a ratio of 10 µL buffer per 5 mg homogenised root tissue. After rotation for 10 min and centrifugation (13 000 rpm, 10 min, 4°C), supernatants were collected and boiled at 60°C for 10 min.

Proteins were separated by SDS‐PAGE using 4%–20% Mini‐PROTEAN TGX Stain‐Free gels (Bio‐Rad) and transferred to 0.45 µm nitrocellulose membranes (Bio‐Rad) with the Trans‐Blot Turbo Transfer System (Bio‐Rad).

Membranes were blocked for 5 min in EveryBlot Blocking Buffer (Bio‐Rad) and incubated for 1 h at room temperature with primary antibodies against mTurquoise2 (Biozol, NBS‐ASJ‐L9MMEL‐60; 1:1000 dilution) in 2.5% TBST with blocking buffer. After three washes in 1× TBST, membranes were incubated with HRP‐conjugated secondary antibody, mouse anti‐goat for mTurquoise2 [Invitrogen, G‐21040; 1:1000 dilution], in TBST with 5% milk for 1 h. Chemiluminescent signals were developed with ECL substrate (Amersham ECL Select or SuperSignal West Femto) and imaged using the ChemiDoc MP Imaging System.

To assess the effect of proteasome inhibition on bHLH104 protein stability, 10‐day‐old *Arabidopsis* seedlings were transferred to liquid Hoagland medium and incubated for 18 h in a plant growth chamber under the same growth conditions described above. Seedlings were treated with either 50 µM MG132 or an equal volume of DMSO (control). After treatment, total protein from roots was extracted and analysed by immunoblotting. Band intensities were quantified using Bio‐Rad Image Lab Software and normalised to total protein staining. For each genotype, three independent biological replicates were used.

### 4.5 Cell‐Free Protein Degradation Assay

The coding sequence of bHLH104 was cloned into an expression vector containing an N‐terminal His tag and transformed into *Escherichia coli Rosetta (DE3)* cells. Single colonies were cultured overnight in LB medium with ampicillin, and secondary cultures were inoculated at 1% volume, grown at 37°C to OD_600_ = 0.6–0.8, and induced with 1 mM IPTG at 28°C for 10–12 h. Cells were harvested by centrifugation and stored at −70°C.

For purification, frozen cell pellets were thawed on ice, resuspended in lysis buffer (30 mM Tris‐HCl, pH 8.0, 150 mM NaCl) supplemented with protease inhibitors (Roche), and sonicated. Lysates were clarified by centrifugation (14 000 rpm, 30 min, 4°C), and His‐tagged bHLH104 protein was purified using a Ni‐NTA column (Cytiva) on a Bio‐Rad NGC chromatography system. Bound proteins were batch‐eluted with 250 mM imidazole in lysis buffer, and fractions containing the target protein were pooled, concentrated using Amicon centrifugal filters, and quantified using absorbance at 280 nm with a NanoQuant plate (Tecan). Protein concentrations were calculated using the molar extinction coefficient predicted by ExPASy ProtParam, and proteins were aliquoted and stored at −70°C.

We followed protocol from Wang et al. (2009), for cell‐free degradation assay. Degradation reactions were initiated by incubating 50 µL of plant extract with purified His‐bHLH104 protein at 22°C. Protein extracts were treated with either 160 µM MG132 or an equal volume of DMSO (control). Reactions were stopped at indicated time points by adding SDS loading buffer and boiling at 95°C for 10 min. Proteins were separated by SDS‐PAGE, transferred to nitrocellulose membranes, and detected by immunoblotting using an HRP‐conjugated anti‐His antibody (Thermo Fisher Scientific, MA1‐21315‐HRP).

### 4.6 Confocal Microscopy

Confocal imaging was performed using a Zeiss LSM 880 laser‐scanning confocal microscope equipped with a 40× C‐Apochromat water immersion objective. Two‐day‐old *A. thaliana* seedlings grown under +Fe conditions were used for imaging. Excitation of mTurquoise2 fluorescence was carried out using a 458 nm laser line, and emitted fluorescence was collected between 493 nm and 613 nm. Images were processed and analysed using the manufacturer's software ZEN lite (Zeiss).

### 4.7 ICP‐MS Analysis

Tissues were harvested, washed sequentially in Tris‐EDTA buffer and deionised water, dried on tissue paper, transferred into pre‐weighed 5‐ml Falcon tubes, and oven‐dried at 65°C for up to 2 weeks. Tissue digestion was performed with 67% nitric acid (ICP‐MS grade) at volumes adjusted to sample mass (1 ml for 20–35 mg, 500 µL for 10–20 mg, 350 µL for < 10 mg). Samples were incubated at room temperature overnight and then heated at 95°C until clear. After centrifugation (4000 rpm, 30 min, 4°C), supernatants were diluted with deionised water to maintain a final acid concentration below 5% (v/v). Elemental analysis was performed using ICP‐MS (Agilent 7700). ICP‐MS measurements were corrected for background signal and normalised by sample dilution factors.

### 4.8 Colorimetric Fe Content Measurement

Seedling Fe content was measured using a BPDS‐based colorimetric assay adapted from Tamarit et al. (2006), as described in Von Der Mark et al. (2021). Whole seedlings were dried overnight at 100°C, ground in an agate mortar (Merck), and weighed. A 10–15 mg aliquot was resuspended in 500 µL of 3% HNO_3_ and incubated for 16 h at 100°C, followed by centrifugation for 5 min at 12 000 rpm. 400 µL of 3% HNO_3_ containing either FeNaEDTA standard or seedling material was mixed with 160 µL of sodium ascorbate (38 mg ml^−1^), 320 µL of the Fe chelator BPDS (1.7 mg ml^−1^), and 126 µL of ammonium acetate solution (diluted 1:3 from saturated solution). The specific absorbance of the Fe–chelator complex was recorded at 535 nm after 5 min, together with a reference measurement at 680 nm for non‐specific absorbance, using a 96‐well plate reader (Infinite 200 PRO, Tecan). The Fe concentration was normalised to the dry weight of the sample. Values from three independent replicates per genotype were averaged, and the SD was calculated.

### 4.9 RNA Isolation and RT‐qPCR Analysis

Total RNA was extracted using the RNeasy Plant Mini Kit (Qiagen) with on‐column DNase digestion. First‐strand cDNA synthesis was performed with the RevertAid Reverse Transcriptase kit (Thermo Fisher Scientific) using 300–500 ng RNA per reaction. RNA was treated with DNase I, followed by heat inactivation with EDTA and oligo(dT) primer annealing. Reverse transcription was carried out at 42°C for 1 h. cDNA was diluted 1:10 and 1:100 for downstream applications and stored at −20°C.

qPCR was performed according to the protocol in Ngigi and Bauer (2023). Each sample was measured in technical duplicates alongside a six‐point standard curve (10^7^–10^2^ copies) for each gene. The PCR programme included an initial denaturation (95°C, 3 min) followed by 40 cycles of 95°C denaturation (10 s), annealing at gene‐specific temperature (15 s), and extension (5 s). Melt curve analysis was conducted from 65°C to 95°C.

Starting quantities were calculated from the standard curves and normalised as described in Ngigi and Bauer (2023). Normalised expression values were visualised as *z*‐score heatmaps using R.

### 4.10 Transgene Copy Number Determination

Relative transgene copy number was determined by qPCR on genomic DNA following the protocol of Ngigi and Bauer (2023). Genomic DNA was extracted from the roots of 10‐day‐old seedlings grown under +Fe conditions. The transgene was amplified with a primer pair spanning the *mTurquoise2–bHLH104* fusion junction, which is specific to the transgene and does not amplify the endogenous bHLH104 locus, and quantified relative to the single‐copy genomic locus EF1Bα2. qPCR was performed as described above, with each sample measured in technical duplicates alongside a six‐point standard curve. Values represent relative transgene amounts across lines from three biological replicates.

### 4.11 RNA Seq and Analysis

RNA‐seq was performed by BMKGENE (Biomarker Technologies). Library preparation and sequencing were conducted according to the service provider's standard protocols. Data were processed following the protocol of Mai (2025).

For DEG analysis, RNA‐seq matrices were analysed in R (v4.3+) with limma (v3.58+) using the limma‐trend workflow appropriate for transcripts per million (TPM) data. Expression values were log_2_‐transformed as log_2_(*x* + 0.5), where *x* is the TPM value. For each comparison (line vs. WT), linear models were fit with empirical Bayes moderation, and contrasts were estimated to obtain log_2_ fold‐changes (log_2_FC) and Benjamini–Hochberg (BH)‐adjusted *p* values. Genes were called differentially expressed if FDR ≤ 0.05 and |log_2_FC| ≥ 0.58 (~1.5‐fold).

### 4.12 Gene Ontology Enrichment

GO Biological Process enrichment was performed with clusterProfiler (v4.10+) against the appropriate org.*.eg.db annotation. Up‐ and down‐regulated DEG sets were tested separately. The gene universe for each test comprised all genes assessed in the corresponding comparison. Over‐representation *p* values were computed by the hypergeometric test and adjusted by BH; terms with FDR ≤ 0.05 were considered significant. When indicated, redundant terms were condensed with simplify() (Wang similarity measure (Wang et al. 2007); cutoff = 0.7), retaining the most significant representative.

### 4.13 Bioinformatic Analyses

Protein domain architecture of bHLH104 was analysed using the MyHits Motif Scan server (https://myhits.sib.swiss/cgi-bin/motif_scan) (Pagni et al. 2007). The bHLH domain was identified at residues 121–181, and the leucine zipper (LZ) domain at residues 181–202. Structures of bHLH104, BTS and their interaction interfaces were predicted with AlphaFold3 (Abramson et al. 2024) using the AlphaFold Server with default settings, which generates five models per job. Model quality was assessed using pLDDT scores for per‐residue confidence and interface predicted Template Modelling scores for interface accuracy.

To assess the contribution of individual residues of the C‐terminal PAA sequence to the predicted bHLH104–BTS interaction, each position was substituted in silico using AlphaFold3, generating five models per sequence. Each of the three C‐terminal residues (P1, A2, A3, N to C) was substituted individually by valine, glycine or alanine, and C‐terminal deletion variants lacking the last one, two or all three residues were also modelled.

The predicted interface was quantified as the iPAE, calculated from the AlphaFold3 PAE output as the mean predicted aligned error over the cross‐chain residue pairs between the motif and a fixed set of BTS interface residues, taken in both directions. The reference set was defined from the WT complex as BTS residues with any heavy atom within 8.0 Å of the WT PAA motif, taken as the union across WT models, and applied unchanged to all variants. The change in iPAE relative to the WT sequence (ΔiPAE) was calculated for each construct, with higher values indicating a weaker predicted interaction. Values were averaged across the five models per construct, and bootstrap confidence intervals were used to estimate uncertainty. Analyses were performed with a custom R pipeline reading PAE values and coordinates from the AlphaFold3 output.

The resulting structures were visualised and analysed using UCSF ChimeraX (Pettersen et al. 2004).

### 4.14 Y2H Assay

Y2H assays were performed as described by Gietz and Schiestl (2007) and Lichtblau et al. (2022). C‐terminal fragments of bHLH104 and b104^dPAA were cloned into the Gateway‐compatible yeast vectors pACT2‐GW and pGBKT7‐GW. Primers used are listed in Supporting Information S24: Table S1. Full‐length BTS, BTSL1 and BTSL2 constructs were described in Lichtblau et al. (2022).

Yeast strain AH109 was co‐transformed with plasmid pairs using the lithium acetate (LiAc) method and selected on synthetic dropout (SD) medium lacking leucine and tryptophan (SD–Leu–Trp). Colonies were cultured overnight in liquid SD–Leu–Trp medium, adjusted to an OD_600_ of 1.0, and spotted (10 µL) in 10‐fold dilution series onto SD medium lacking leucine, tryptophan, and histidine (SD–Leu–Trp–His). To suppress autoactivation, SD–Leu–Trp–His plates supplemented with 15 mM 3‐amino‐1,2,4‐triazole were used specifically for assays involving BTSL2. Plates were incubated at 30°C, and growth was monitored for up to 14 days.

### 4.15 UbiGate Assay

Ubiquitination assays were performed using the UbiGate system as described previously (Kowarschik et al. 2018). Constructs were assembled using Golden Gate assembly. The following modules were assembled into pGG‐pET28 destination vector: 6×His‐tagged ubiquitin, E1 ligase (*A. thaliana* UBA1), E2 ligase (*A. thaliana* UBC8), E3 ligase (BTS‐HC), substrate (BHLH105 and BHLH104 coding sequences). The coding sequences were codon‐optimised for *E. coli* expression using GeneArt Gene Optimiser software and synthesised by GeneArt Gene Synthesis (Thermo Fisher Scientific). Primers used are listed in Supporting Information S24: Table S1. Assembled vectors were transformed into *E. coli Rosetta (DE3)* and expressed by induction with 1 mM IPTG and 3% ethanol at 28°C for 3 h. Cells were harvested by centrifugation and stored at −80°C. Protein extraction and sample preparation were performed as previously described in Ortmann et al. (2023).

Proteins were separated by SDS‐PAGE using TGX precast gels (Bio‐Rad) and transferred to 0.2 µm nitrocellulose membranes with the Trans‐Blot Turbo system (Bio‐Rad). Membranes were blocked with 5% (w/v) non‐fat dry milk in TBST and probed with the following primary antibodies: HRP‐conjugated anti‐Myc (Thermo Fisher Scientific, R951‐25‐HRP), anti‐GST (clone 8‐326, Thermo Fisher Scientific), followed by HRP‐conjugated secondary antibody (Jackson ImmunoResearch), HRP‐conjugated Strep‐Tactin (Thermo Fisher Scientific), and HRP‐conjugated anti‐6×His (Thermo Fisher Scientific, MA1‐21315‐HRP). Signals were detected using the Amersham ECL Prime kit (Cytiva) and visualised with the ChemiDoc MP Imaging System (Bio‐Rad).

### 4.16 Statistical Analysis and Data Visualisation

All statistical analyses and data visualisation were performed using RStudio (version 2025.04.1). Phenotyping data were visualised using ggplot2. Gene expression data were *Z*‐score normalised using the scale() function in R, and gene expression heatmaps were created using the pheatmap package to visualise normalised expression levels across tissues and experimental conditions. For ICP‐MS data visualisation, the ggPlantMap package (Jo and Kajala 2024) was used.

Statistical comparisons were performed using two‐way ANOVA followed by Tukey's Honest Significant Difference test for multiple comparisons. A *p*‐value threshold of 0.05 was considered significant.

For the analysis of phenotype severity across *pb104::b104^dPAA* lines (Supporting Information S3: Figure S3), Spearman rank correlations were calculated between primary root length and relative transgene copy number, transgene transcript level, b104^dPAA protein abundance and iron content, across the eight independent transgenic lines (WT excluded). In the accompanying heatmap, each parameter was expressed as a *z*‐score across WT and the eight lines. Significance thresholds were **p < 0.01; *p < 0.05.

## Supporting information


**Figure S1:** mTurquoise2‐tagged b104^dPAA complements the *bhlh104‐1* phenotype under Fe deficiency. Representative images of 12‐day‐old seedlings grown on iron‐deficient (‐Fe) medium. Left panel: wild‐type Col‐0 and *bhlh104‐1*. Right panel: *pb104::mTurq2‐b104^dPAA*/*bhlh104‐1* and *pUBQ10::mTurq2‐b104^dPAA*/*bhlh104‐1* complementation lines. The *bhlh104‐1* displays chlorotic leaves compared to Col‐0 under Fe deficiency. Expression of mTurquoise2‐tagged b104^dPAA under either the native *BHLH104* promoter or the *UBQ10* promoter restores leaf health in the *bhlh104‐1* background, showing that the mTurquoise2‐tagged protein is biologically functional. Scale bars: 2 cm.

**Figure S2:** Preliminary phenotypic screen of independent transgenic lines and selection of *pb104::b104^dPAA* lines for analysis. (A) 26‐day‐old plants of Col‐0 wild type (WT), *pb104::b104* (four independent lines), *pb104::b104^dPAA* (eight independent lines) and *pUBQ10::b104* (four independent lines), grown under the same conditions. Each tray contained eight plants derived from an independent T1 transgenic line. The eight *pb104::b104^dPAA* lines used in the initial characterisation experiment are enclosed in the black rectangle. Lines selected for detailed phenotyping are indicated in bold: *pb104::b104* lines #1 and #2, *pb104::b104^dPAA* lines #1–#4, and *pUBQ10::b104* lines #1 and #2. Scale bar = 2 cm. (B) Overview of the *pb104::b104^dPAA* lines and the analyses in which each was used. The eight independent lines were propagated to the T3/T4 generation, selected on hygromycin and genotyped by PCR (see Materials and Methods), and grouped by phenotype severity (strong: #1, #2, #5, #6; weak: #3, #4, #7, #8). All eight lines were used for initial characterisation (Supporting Figure S3). Four lines (#1–#4) were then selected for detailed phenotyping, and two lines (#2 and #3) were used for RNA‐seq and ICP‐MS analysis. Ticks indicate the analyses performed for each line.

**Figure S3:** Phenotype severity across independent *pb104::b104^dPAA* lines correlates with transgene copy number, b104^dPAA protein abundance and iron content, but not with transgene transcript level. Eight independent transgenic lines carrying *pb104::b104^dPAA* (b104^dPAA expressed under the native *BHLH104* promoter) were analysed together with the untransformed Col‐0 wild type (WT). Lines are ordered by phenotype severity as judged from the parental plants in Supporting Figure S2. All measurements were performed on plants grown under iron‐sufficient (+Fe) conditions. Lines selected for detailed phenotyping are indicated in bold. (A) Spearman rank correlation of relative transgene copy number, transgene transcript level, b104^dPAA protein abundance and iron content with primary root length, across the eight independent transgenic lines (n = 8 lines; WT excluded from the correlation). In the heatmap, each parameter (row) is shown as a z‐score across WT and the eight lines (orange, above the row mean; blue, below; white, near the mean). The right‐hand column gives the Spearman coefficient (ρ) of each parameter against primary root length, which serves as the reference row (ref). Significance: *** p < 0.001, ** p < 0.01, * p < 0.05, ns not significant (p ≥ 0.05). (B) Relative transgene copy number, determined by quantitative PCR on genomic DNA following the protocol of (Ngigi and Bauer 2023). The transgene was amplified with a primer pair spanning the mTurquoise2–bHLH104 fusion junction, which is specific to the transgene and does not amplify the endogenous bHLH104 locus, and quantified relative to the single‐copy genomic locus EF1Bα2 (mean ± SD, n = 3). (C) Transgene transcript level in roots of 10‐day‐old seedlings grown under +Fe, determined by RT‐qPCR using the same mTurquoise2–bHLH104 transgene amplicon as in (B), with quantification and normalisation performed as described in Materials and Methods (mean ± SD, n = 3). (D) b104^dPAA protein abundance in roots of 10‐day‐old seedlings grown under +Fe, detected by immunoblotting with an antibody against mTurquoise2; band intensities were quantified with Image Lab (Bio‐Rad) and normalised to total protein staining (mean ± SD, n = 3). (E) Iron content of whole seedlings, measured by a BPDS‐based colorimetric assay adapted from Tamarit et al. (2006), as described in von der Mark et al. (2021). Dried tissue was digested in 3% HNO_3_, and the Fe‐BPDS complex was measured at 535 nm against a 680 nm reference on a 96‐well plate reader; iron concentration was normalised to sample dry weight (mean ± SD, n = 3). (F) Primary root length of 12‐day‐old seedlings grown on modified half‐strength Hoagland medium under +Fe (mean ± SD, n = 12 seedlings per line).

**Figure S4:** Whole‐plant phenotypes of *pb104::b104^dPAA* lines. Quantitative analysis of aboveground phenotypes using the Phenospex PlantEye system: (A) 3D leaf area (mm^2^); (B) NDVI (Normalized Difference Vegetation Index). (C) Flowering time, recorded as days to bolting. (D) Seed number per plant, estimated from total seed weight based on mean weight per 100 seeds. Data represent means ± SD (n = 7–12 biological replicates per line). Statistical analysis was performed using two‐way ANOVA followed by Tukey's post hoc test. Different letters indicate statistically significant differences at p < 0.05.

**Figure S5:** Response of b104^dPAA transgenic lines to iron‐limiting conditions in alkaline calcareous soil (ACS). *Arabidopsis thaliana* transgenic lines were grown in either control soil (pH 5.9) or alkaline calcareous soil (ACS, pH 8.1).(A) Photos of four‐week‐old plants grown in control and ACS soil conditions. (B) List of plant lines used in this experiment, numbered from L1 to L7. Scale bar = 2 cm. (C) 3D leaf area measurements: Plants were evaluated for leaf expansion and overall growth in both conditions. (D) NDVI (Normalized Difference Vegetation Index): NDVI values were measured to assess photosynthetic activity in response to ACS conditions. (E) Flowering time: Flowering time was compared between control and ACS conditions, with letters indicating statistical differences. Statistical significance was assessed by two‐way ANOVA followed by Tukey's HSD test (p < 0.05). n = 8 plants per line.

**Figure S6:** Constitutive expression of bHLH104 did not alter growth and physiological traits in *Arabidopsis*. (A) Schematic representation of the construct used to generate transgenic lines expressing full‐length bHLH104 (*pUBQ10::bHLH104*). The construct is driven by the ubiquitin 10 (*UBQ10*) promoter and is fused at the N‐terminus to the mTurquoise2 fluorescent tag. Scale bar = 200 bp. (B‐C) Photos and root length quantification data of 12‐day‐old seedlings grown vertically on Hoagland medium supplemented with iron (+Fe). Scale bar = 2 cm. (D) Representative images of 4‐week‐old *Arabidopsis* plants grown in soil. Shown are Col‐0 (WT) and two independent *pUBQ10::b104* lines (#1, #2). Scale bar = 2 cm. (E–F) Quantitative analysis of aboveground phenotypes acquired using the Phenospex PlantEye system: (E) 3D leaf area (mm^2^); (F) NDVI (Normalized Difference Vegetation Index). (G) Flowering time, measured manually as the number of days to bolting. Data represent means ± SD (n = 7–12 biological replicates per line). Statistical analysis was performed using two‐way ANOVA followed by Tukey's post hoc test. Different letters indicate statistically significant differences at p < 0.05.

**Figure S7:** Elemental content analysis of Mn, Zn, and Cu in 10‐day old *Arabidopsis* seedlings. Heatmap showing the distribution of Mn, Zn, and Cu content (in µg/g dry weight) across different *Arabidopsis* seedlings, including wild‐type (WT), *pb104::b104* (lines #1, #2), *pb104::b104^dPAA* (lines #2, #3), *pUBQ10::b104* (lines #1, #2). The colour scale indicates the range of elemental concentrations from low (light blue) to high (dark blue). Seedlings were grown in Hoagland media with or without 50 µM Fe supplementation. Data were collected from 10‐day‐old seedlings, with elemental content measurements obtained via ICP‐MS analysis. Three biological replicates per line were used. The plot was generated using the ggPlantMap package in RStudio. Asterisks indicate genotypes that differ significantly from WT within the same tissue and treatment condition (p < 0.05), as determined by two‐way ANOVA and Tukey's post hoc test (n = 3 for each group).

**Figure S8:** Differential expression and GO enrichment across seedling tissues. (A) Counts of differentially expressed genes (DEGs) for each line vs WT in root and shoot under Fe(+) and Fe(−). Orange = up‐regulated; blue = down‐regulated; numbers above/below bars indicate counts for each category. Total DEGs for each condition are the sum of up‐regulated and down‐regulated counts. DEGs were defined at FDR ≤ 0.05 and |log2FC | ≥ 0.58. X‐axis labels show the abbreviated line IDs. (B) GO Biological Process enrichment for the up‐regulated (left panel) and down‐regulated (right panel) DEG sets. Columns correspond to tissue/Fe combinations (Root Fe(+), Root Fe(−), Shoot Fe(+), Shoot Fe(−)). Each dot represents a significant term (BH FDR ≤ 0.05); dot size = number of genes in the term, fill = −log10(FDR), and ring colour = GeneRatio. Terms plotted are the top‐ranked per panel; the gene universe was all genes tested in the corresponding comparison.

**Figure S9:** Expression profiles of 23 selected Fe homeostasis genes involved in Fe acquisition and mobilisation (A) Plant lines used in this experiment. (B) Clustered heatmap showing expression profiles of 23 Fe homeostasis genes (gene selection based on Ngigi et al. 2025; Schwarz and Bauer 2020) obtained using RNA sequencing in root and shoot tissues under +Fe and ‐Fe conditions. *Arabidopsis* seedlings were grown for 10 days after sowing (DAS) under either iron‐sufficient (+Fe) or iron‐deficient (‐Fe) conditions. (C) Clustered heatmap showing expression profiles of 23 Fe homeostasis genes (gene selection based on Ngigi et al. 2025; Schwarz and Bauer 2020) obtained using RNA sequencing in young leaves, old leaves, and roots under both +Fe and ‐Fe conditions. *Arabidopsis* plants were grown hydroponically under iron‐sufficient (+Fe) conditions for 27 days, followed by a 3‐day treatment with either +Fe or ‐Fe media. Heatmap was generated in RStudio version 4.4.1 using calculated Z‐scores of normalised gene expression values. Purple indicates low expression and yellow indicates high expression.

**Figure S10:** Targeted gene expression profiling in seedlings. (A) Schematic representation of the experimental design. *Arabidopsis* seedlings were grown for 10 days after sowing (DAS) under either iron‐sufficient (+Fe) or iron‐deficient (‐Fe) conditions. Shoot and root tissues were collected separately for RT‐qPCR analyses. (B) Genotypes used in this study. Abbreviations of lines used: L1, wild‐type Col‐0; L2, pb104::mTurquoise2‐bHLH104 line #1; L3, pb104::mTurquoise2‐bHLH104 line #2; L4, pb104::mTurquoise2‐b104^dPAA line #2; L5, pb104::mTurquoise2‐b104^dPAA line #3; L6, pUBQ10::mTurquoise2‐bHLH104 line #1; L7, pUBQ10::mTurquoise2‐bHLH104 line #2. (C) Quantification of mTurq2‐tagged bHLH104 transgenes in indicated plant lines using RT‐qPCR Letters indicating statistical differences. Statistical significance was assessed by two‐way ANOVA followed by Tukey's HSD test (p < 0.05). n = 3 per group. (D) Clustered heatmaps showing the expression profiles of selected Fe homeostasis genes obtained using RT‐qPCR. Heatmaps were generated in RStudio version 4.4.1 using calculated Z‐scores of normalised gene expression values across root and shoot tissues for both +Fe and ‐Fe conditions. Purple indicates low expression and yellow indicates high expression.

**Figure S11:** Elemental content analysis of Mn, Zn and Cu in mature *Arabidopsis thaliana*. Heatmap showing the distribution of manganese (Mn), zinc (Zn) and copper (Cu) content (in µg/g dry weight) in young leaves, old leaves and roots of *Arabidopsis* transgenic lines, including wild type (WT), *pb104::b104* (lines #1, #2), *pb104::b104^dPAA* (line #3) and *pUBQ10::b104* (lines #1, #2). Plants were grown hydroponically under iron‐sufficient (+Fe) conditions for 27 days, followed by a 3‐day treatment with either +Fe or ‐Fe medium. Separate colour scales are shown for each element, with darker blue shades representing higher concentrations. Metal concentrations were measured by ICP‐MS. Data represent mean values from three biological replicates (n = 3). The plot was generated using the ggPlantMap package in RStudio. Asterisks indicate genotypes that differ significantly from WT within the same tissue and treatment condition (p < 0.05), as determined by two‐way ANOVA and Tukey's post hoc test (n = 3 for each group). The tissue layout for reading the heatmap is shown as a legend in the figure.

**Figure S12:** Differential expression and GO enrichment across reproductive stage tissues. (A) Numbers of differentially expressed genes (DEGs) for each line vs WT in root (Root), young leaves (YL), and old leaves (OL) under Fe(+) and Fe(−). Orange bars show up‐regulated DEGs; blue bars show down‐regulated DEGs; values above/below bars are counts for each category. Total DEGs for each condition are the sum of up‐regulated and down‐regulated counts. DEGs were defined at FDR ≤ 0.05 and |log2FC | ≥ 0.58. (B) GO Biological Process enrichment for the up‐regulated (left panel) and down‐regulated (right panel) DEG sets. Columns correspond to tissue/Fe combinations (Root, YL, OL × Fe(+), Fe(−)). Each dot represents a significant term (BH FDR ≤ 0.05); dot size = number of genes in the term, fill = −log10(FDR), and ring colour = GeneRatio. Terms shown are the top‐ranked per panel; the gene universe comprised all genes tested in the corresponding comparison. Abbreviations: YL, young leaves; OL, old leaves.

**Figure S13:** Targeted gene expression profiling in reproductive‐stage plants. (A) Plant lines used in this experiment. Abbreviations of lines used: L1, wild‐type Col‐0; L2, pb104::mTurquoise2‐bHLH104 line #1; L3, pb104::mTurquoise2‐bHLH104 line #2; L4, pb104::mTurquoise2‐b104^dPAA line #2; L5, pb104::mTurquoise2‐b104^dPAA line #3; L6, pUBQ10::mTurquoise2‐bHLH104 line #1; L7, pUBQ10::mTurquoise2‐bHLH104 line #2. (B) Experimental design for the dataset shown. *Arabidopsis* plants were grown hydroponically under iron‐sufficient (+Fe) conditions for 27 days, followed by a 3‐day treatment with either +Fe or ‐Fe media. (C) Schematic representation of tissue separation used for RT‐qPCR analysis. Young and old leaves were separated from the roots to examine tissue‐specific iron accumulation and gene expression. (D) Clustered heatmaps showing the expression profiles of selected Fe homeostasis genes conditions obtained using RT‐qPCR in young leaves, old leaves, and roots under both +Fe and ‐Fe. Data represent the mean of three biological replicates (n = 3). Z‐scores were calculated from normalised RT‐qPCR expression values and visualised in RStudio version 4.4.1. Yellow indicates high expression; purple indicates low expression.

**Figure S14:** Tissue‐specific metal accumulation in dPAA line #2 grown under iron‐deficient conditions. (A) Experimental setup: Col‐0 and *pb104::b104^dPAA* line #2 seedlings were grown hydroponically in Fe‐deficient (‐Fe) media for 27 days, followed by a 3‐day treatment with either +Fe or ‐Fe media. Tissue collection occurred at 30 days after sowing (DAS).(B) Tissue layout: Young leaves, old leaves, and roots were separated to examine tissue‐specific metal accumulation (Fe, Mn, Zn, Cu).(C) Heatmap of metal content: The distribution of Fe, Mn, Zn, and Cu content (in µg/g dry weight) in dPAA line #2 and Col‐0. The colour scale reflects metal concentrations, with darker blue shades indicating higher content. *pb104::b104^dPAA* line #2 exhibited significant Fe accumulation in young leaves after Fe resupply, in contrast to Col‐0, where most Fe remained in the roots. Data represent the mean values from three biological replicates (n = 3). Elemental content was measured by ICP‐MS. The plot was generated using the ggPlantMap package in RStudio. Asterisks indicate genotypes that differ significantly from WT within the same tissue and treatment condition (p < 0.05), as determined by two‐way ANOVA and Tukey's post hoc test (n = 3 for each group).

**Figure S15:** Expression profiles of genes involved in Mn, Zn, and Cu homeostasis (A) Plant lines used in this experiment. (B) Clustered heatmap showing expression profiles from RNA sequencing experiment of genes involved in manganese (Mn), zinc (Zn), and copper (Cu) homeostasis across root and shoot tissues under +Fe and ‐Fe conditions. *Arabidopsis* seedlings were grown for 10 days after sowing (DAS) under either iron‐sufficient (+Fe) or iron‐deficient (‐Fe) conditions. (C) Clustered heatmap showing expression profiles from RNA sequencing experiment of genes involved in manganese (Mn), zinc (Zn), and copper (Cu) homeostasis in young leaves, old leaves, and roots under both +Fe and ‐Fe conditions. *Arabidopsis* plants were grown hydroponically under iron‐sufficient (+Fe) conditions for 27 days, followed by a 3‐day treatment with either +Fe or ‐Fe media. Genes include metal transporters and the Cu homeostasis regulator *SPL7*. Heatmap was generated in RStudio version 4.4.1 using calculated Z‐scores of normalised gene expression values. Purple indicates low expression and yellow indicates high expression.

**Figure S16:** Additional independent replicates and anti‐mTurq immunoblots for detection of mTurquoise2‐bHLH104 and ‐b104^dPAA protein in various transgenic lines. Genotypes and transgenic lines used in this experiment. (A) Genotypes and transgenic lines used in this experiment. The mTurquoise2 fluorescent tag is fused at the N‐terminus of bHLH104 and b104^dPAA in all transgenic lines. (B) Experiment 1: immunoblot of total protein extracted from roots of 10‐day‐old seedlings (see also main text figure, Figure 4B). (C) Experiment 2: immunoblot of total protein extracted from 10‐day‐old whole seedlings. (D) Experiment 3: immunoblot of total protein extracted from 6‐day‐old whole seedlings. For each experiment, top panels show the anti‐mTurquoise2 blot and bottom panels show total protein stain as loading control. Molecular weight markers (kDa) are indicated on the right of each blot. Red arrowheads indicate the expected band position for the full‐length mTurquoise2‐bHLH104 fusion protein (~70 kDa). Lower molecular weight bands detected by the anti‐mTurquoise2 antibody likely represent truncated or partially degraded forms of the mTurquoise2‐tagged protein. Across all three independent experiments, mTurquoise2‐b104^dPAA protein is consistently more abundant than mTurquoise2‐bHLH104, supporting that deletion of the PAA sequence stabilises bHLH104 protein. The three experiments represent independent biological samples differing in seedling age (6 or 10 days) and tissue type (whole seedlings or roots), demonstrating the robustness of the observed protein accumulation pattern.

**Figure S17:** Quantification of His‐b104 and Actin band intensities at selected time points of the cell‐free degradation assay shown in Figure 4D. WT extract was incubated with purified His‐b104 protein in the presence of DMSO (vehicle) or MG132 (proteasome inhibitor) and sampled at different time points as indicated. (A) Relative His‐b104 protein band levels, quantified and normalised to total lane protein, expressed as a percentage of the 0 min signal for each treatment. (B) Relative Actin levels at the corresponding time points, normalised to total lane volume and expressed as a percentage of the 0 min signal. Dashed line indicates the 0 min (100%) reference. In the DMSO series the Actin signal remained close to the 0 min level while His‐b104 declined, indicating that the loss of His‐b104 signal is not accounted for by a general reduction in extract protein.

**Figure S18:** Yeast two‐hybrid assay results showing protein–protein interactions between bHLH104, BTSL1 and BTSL2. bHLH104 interacts with BTSL1 and BTSL2, but not b104^dPAA. b104^dPAA retains the ability to heterodimerise with full‐length bHLH104, serving as a control for the presence and expression of the PAA‐deleted variant. Full length bHLH104 shows autoactivation in Y2H. Hence bHLH104‐C and bHLH104‐C^dPAA were used. Yeast co‐transformed with the AD and BD combinations were spotted in 10‐fold dilution series (A600 = 10–1–10–4) on SD‐LW (transformation control) and SD‐LWH plates supplemented with different 3AT concentrations (conc.) as indicated (selection for protein interaction). The data in Figure 5E and Supporting Figure S18 derive from a single yeast two‐hybrid experiment in which all combinations were transformed, plated and imaged in parallel. The empty vector controls bHLH104‐C + Empty, Empty + bHLH104‐C, b104‐C^dPAA + Empty and Empty + b104‐C^dPAA are therefore shown as the same images in both figures. Negative controls: empty vectors. Black arrows indicate interaction.

**Figure S19:** Independent replicates of the UbiGate ubiquitination assay for bHLH104. Anti‐Strep immunoblots from three independent UbiGate experiments (Experiment 1, shown in Figure 6B; Experiments 2 and 3) supporting PAA‐dependent ubiquitination of bHLH104 by BTS. The anti‐Strep antibody detects the Strep‐tagged substrate (~40 kDa; brown arrows, non‐ubiquitinated substrate). Green arrows indicate a distinct ~70 kDa band, present only in the bHLH104‐C sample with all components present and absent from all control samples and from the bHLH104‐C^dPAA samples, consistent with polyubiquitinated bHLH104‐C (a shift of ~30 kDa, corresponding to the addition of three to four ubiquitin moieties). Grey arrows indicate additional higher‐molecular‐weight bands that are not considered specific, as similar bands are present across control lanes. The ~70 kDa band was reproduced in all three experiments, supporting the result shown in Figure 6B.

**Figure S20:** Immunoblots confirming expression of all proteins for ubiquitination activity of the E3 ligase in the UbiGate assay. All panels are from the same membrane, sequentially probed with different antibodies after stripping. Anti‐GST detects the E2 ligase (GST‐UBC8); anti‐Myc detects the E3 ligase (Myc‐BTS‐HC); anti‐His‐Ub detects total ubiquitin conjugates (His‐Ub). The anti‐His‐Ub blot detects all ubiquitin conjugates present in the reaction, including autoubiquitinated E2 and E3. The assessment of substrate ubiquitination is therefore based on the anti‐Strep blot shown in Figure 6B. The violet bracket indicates autoubiquitinated E2 ligase; the blue bracket indicates autoubiquitinated E3 ligase. Bands in the control lanes of this blot represent E2 and E3 autoubiquitination and are independent of substrate ubiquitination. Lane assignments are as in Figure 6B.

**Figure S21:** UbiGate assay testing ubiquitination of bHLH115 by BTS‐HC. UbiGate assay testing whether bHLH115 is ubiquitinated by BTS‐HC in *E. coli* cells. All four panels are from the same experiment, probed sequentially after stripping: anti‐GST (E2 ligase UBC8, purple box), anti‐Myc (E3 ligase BTS‐HC, blue box), anti‐Strep (substrate bHLH115, green box) and anti‐His‐Ub (ubiquitin, red box). Lane compositions are indicated above, with +/− denoting the presence or absence of E2 (UBC8), E3 (BTS‐HC) and substrate (Strep‐tagged bHLH115); the first lane contains E2 and E3 without substrate as a substrate‐negative control. The anti‐Strep blot (green box) reports the substrate: brown arrows indicate the unmodified bHLH115, and green arrows indicate higher‐molecular‐weight bands in the lane where E2, E3 and substrate were all present, consistent with polyubiquitinated bHLH115. Some higher‐molecular‐weight bands are also present in control lanes and are not considered specific. The anti‐His‐Ub blot (red box) detects all ubiquitin conjugates present in the reaction, including autoubiquitinated E2 and E3. We therefore base the assessment of substrate ubiquitination on the anti‐Strep blot. The violet bracket indicates autoubiquitinated E2 ligase (UBC8) and the blue bracket autoubiquitinated E3 ligase (BTS‐HC). These results are consistent with the previously reported role of bHLH115 as a BTS target (Selote et al. 2015; Xing et al. 2021) and are presented as supporting evidence for the suitability of the UbiGate system.

**Figure S22:** Validation of Fe deficiency treatment in wild‐type plants. Expression of Fe deficiency marker genes in wild‐type Col‐0 roots and shoots under Fe‐sufficient (+Fe) and Fe‐deficient (‐Fe) conditions. RNA‐seq data are presented as transcripts per million (TPM). *IRT1* and *FRO2*, encoding the root Fe uptake transporter and ferric reductase, respectively, show higher expression under ‐Fe in roots, although the difference was not statistically significant due to replicate variability. *BHLH39* is induced under ‐Fe compared to +Fe in both roots and shoots. *BHLH100* is induced under ‐Fe compared to +Fe predominantly in shoots. These expression patterns confirm that the Fe deficiency treatment elicited the expected transcriptional ‐Fe response in wild‐type plants. Data represent mean ± SD (n = 3 biological replicates). Statistical analysis was performed using one‐way ANOVA followed by Tukey's HSD test (p < 0.05). Different letters indicate statistically significant differences. Note that in the main figure heatmaps (Figure 2), these differences are not visible due to Z‐score normalisation being dominated by high expression values in *pb104::b104^dPAA* lines.

**Figure S23:** Original blots for Figure 4B. Red arrows indicate the expected band position for the mTurquoise2‐bHLH104 fusion protein.

**Table S1:** Primers used in this study.

## Data Availability

The data that support the findings of this study are openly available in ArrayExpress at https://www.ebi.ac.uk/biostudies/arrayexpress, reference number E‐MTAB‐16555. Confocal microscopy images are openly available in the BioImage Archive at https://www.ebi.ac.uk/biostudies/bioimages, accession number S‐BIAD2765.
