# Supplementary material for: A Three Amino Acid Sequence Gates Protein Stability Control of bHLH104 and Iron Uptake in Arabidopsis thaliana
Source: Plant Cell Environ. 2026 Sep 22;49(12):10421–41. doi: 10.1111/pce.70879 (PMC13725251; doi:10.1111/pce.70879)
Supplement: Supplementary file 1 [file PCE-49-10421-s012.pdf]

Supplementary Table 1. Primers used in this study.

fw = forward, rev = reverse, d = delta a.k.a. deletion, -C = C-term, (att)B1/B2 = Gateway B attachment sites for BP reaction.

Primer name	Sequence	Purpose	Origin
**Amplification of full-length CDS or deletion constructs for Y2H**			
bHLH104-C_B1 fw	GGGGACAAGTTTGTACAAAAAAGCAGGCTTCATGGCTCT	cloning of bHLH104-C (attB1)	lichtblau 2023
bHLH104-C-d_B2 rev	GGGGACCACTTTGTACAAGAAAGCTGGGTCTTAAGGCCTGAGTTCTTGAT	cloning of bHLH104-C-d (attB2)	This study
bHLH104_B2 rev	GGGGACCACTTTGTACAAGAAAGCTGGGTCTTAAGCAGCAGGAGGCCTG	cloning of bHLH104-C (attB2)	lichtblau 2022
**Cloning bHLH104 promoter and CDS for transgenic lines**			
bHLH104 fw	aacaggtctcaggctggatgtatccttctctcgacg	Cloning of bHLH104/bHLH104-d CDS	This study
bHLH104 rev	aacaggtctcactgaAGCAGCAGGAGGCCTGA	Cloning of bHLH104 CDS	This study
bHLH104-d rev	aacaggtctcactgaAGGCCTGAGTTCTTGAT	Cloning of bHLH104-d CDS	This study
pbHLH104 fw	AACAGGTCTCAACCTCTAGCTCTGAGAAGCGGCATC	Cloning of bHLH104 promoter	
pbHLH104 rev	AACAGGTCTCATGTTTTGAGAGTCTCCAAACAAATT	Cloning of bHLH104 promoter	This study
**Amplification of full-length CDS or deletion constructs for Ubigate**			
BTS-HC fw	ttgaagacaaaATGGCAGAACG	Cloning BTS HC fragment	This study
BTS-HC_rev	ttgaagacaaaagcTTAGCTGCTGGTG	Cloning BTS HC fragment	This study
optbhlh104-C fw	GGGGACAAGTTTGTACAAAAAAGCAGGCTTCATGACCGATAAACCGG	Cloning bHLH104 in pUGTP1 (attB1)	This study
optbhlh104 rev	GGGGACCACTTTGTACAAGAAAGCTGGGTCTTATGCTGCAGGCGGA	Cloning bHLH104 in pUGTP1 (attB2)	This study
optbhlh104d rev	GGGGACCACTTTGTACAAGAAAGCTGGGTCTTACGGACGCAGTTCT	Cloning bHLH104 in pUGTP1 (attB2)	This study
**RT-qPCR primers**			
EFc_fw	TATGGGATCAAGAAACTCACAAT	for RTqPCR	
EFc_rev	CTGGATGTACTCGTTGTTAGGC	for RTqPCR	
EFg_fw	TCCGAACAATACCAGAACTACG	for RTqPCR	
EFg_rev	CCGGGACATATGGAGGTAAG	for RTqPCR	
FIT_fw	CCCTGTTTCATAGACGAGAAC	for RTqPCR	
FIT_rev	ATCCTTCATACGCCCTCTCC	for RTqPCR	
IRT1_fw	AAGCTTTGATCACGGTTGG	for RTqPCR	
IRT1_rev	TTAGGTCCCATGAACTCCG	for RTqPCR	
FRO2_fw	CTTGGTCATCTCCGTGAGC	for RTqPCR	
FRO2_rev	AAGATGTTGGAGATGGACGG	for RTqPCR	
BHLH39_fw	GACGGTTTCTCGAAGCTTG	for RTqPCR	
BHLH39_rev	GGTGGCTGCTTAACGTAACAT	for RTqPCR	
BTS_fw	CGGGGAAGGACTAGGAATCG	for RTqPCR	
BTS_rev	CAGCAGATGGGGCAATTTGT	for RTqPCR	
PYE_fw	GTTCCCAGGACTTCCCATTT	for RTqPCR	
PYE_rev	GTGTCTGGGGATCAGGTTGT	for RTqPCR	
BHLH104_fw	GGTTGAGGAGGGAGAAGCTAAATG	for RTqPCR	
BHLH104_rev	ACGGATTGCATCATCGAGTATAGC	for RTqPCR	
ILR3_fw	GCATGTAGAGAGAAGCAGCGAC	for RTqPCR	
ILR3_rev	TGCGGACAGCATCAACCAAG	for RTqPCR	
BHLH34_fw	AATCCGGGTTGTGAATCAG	for RTqPCR	
BHLH34_rev	CTTCTCCGCCTTCAACAC	for RTqPCR	
BHLH115_fw	TGAATCAAGCAAGAGATGAA	for RTqPCR	
BHLH115_rev	CTGTTTCTCATCACGCAGC	for RTqPCR	
BTSL1_fw	CTCCCCAGTGAAGGCTCTTC	for RTqPCR	
BTSL1_rev	GACCTGCATATCTCCAAGCGA	for RTqPCR	
BTSL2_fw	GCTTGTATGTCGCGACTCAT	for RTqPCR	
BTSL2_rev	AGAGCCTTCACCGGAGAATT	for RTqPCR	
URI_fw	GGACTCCAACGAAGTTGC	for RTqPCR	
URI_rev	GTCTTCTCTGGCCTTTGC	for RTqPCR	
FER1_fw	TAAGCCACTACTCCCTCACG	for RTqPCR	
FER1_rev	TTGTTTGTGTCCACCGTAGC	for RTqPCR	
IMA1_fw	TGCTTCCACCGTGTATGTTG	for RTqPCR	
IMA1_rev	CAGGAGCATAATCATAGCCACTG	for RTqPCR	
mTurq2_fw	GTCCAAGCTGAGCAAAGACC	for RTqPCR	
mTurq2_rev	CTTGTACAGCTCGTCCATGC	for RTqPCR	
BHLH100_fw	GATGCATAGAGTAAAAGAGTCGCT	for RTqPCR	
BHLH100_rev	CCTCCCACCAATCAAACG	for RTqPCR	
BHLH11_fw	TCCATGCCTCTTCTTTAGC	for RTqPCR	
BHLH11_rev	GGAACTATCTTGAACAGCTTG	for RTqPCR	
